# Approaching cumulative quintuple bonds in all-metal uranyl analogs

**DOI:** 10.1093/nsr/nwag462

**Published:** 2026-07-30

**Authors:** Xingxing Gong, Jiaxin Chen, Xinyu Liu, Ningxin Jiang, Jingxiang Zou, Yichi Zhang, Yue Zhao, Shuao Wang, Zhenyang Lin, Congqing Zhu

**Affiliations:** State Key Laboratory of Coordination Chemistry, Jiangsu Key Laboratory of Advanced Organic Materials, School of Chemistry and Chemical Engineering, Nanjing University, Nanjing 210023, China; Department of Chemistry, The Hong Kong University of Science and Technology, Clear Water Bay, Kowloon, Hong Kong 999077, China; State Key Laboratory of Coordination Chemistry, Jiangsu Key Laboratory of Advanced Organic Materials, School of Chemistry and Chemical Engineering, Nanjing University, Nanjing 210023, China; State Key Laboratory of Coordination Chemistry, Jiangsu Key Laboratory of Advanced Organic Materials, School of Chemistry and Chemical Engineering, Nanjing University, Nanjing 210023, China; Laboratory of AI for Electrochemistry (AI4EC Lab), Tan Kah Kee Innovation Laboratory (IKKEM), Xiamen 361005, China; Department of Chemistry, The Hong Kong University of Science and Technology, Clear Water Bay, Kowloon, Hong Kong 999077, China; State Key Laboratory of Coordination Chemistry, Jiangsu Key Laboratory of Advanced Organic Materials, School of Chemistry and Chemical Engineering, Nanjing University, Nanjing 210023, China; State Key Laboratory of Radiation Medicine and Protection, School for Radiological and Interdisciplinary Sciences (RAD-X) and Collaborative Innovation Center of Radiation Medicine of Jiangsu Higher Education Institutions, Soochow University, Suzhou 215123, China; Department of Chemistry, The Hong Kong University of Science and Technology, Clear Water Bay, Kowloon, Hong Kong 999077, China; State Key Laboratory of Coordination Chemistry, Jiangsu Key Laboratory of Advanced Organic Materials, School of Chemistry and Chemical Engineering, Nanjing University, Nanjing 210023, China

**Keywords:** metal–metal bond, cumulative quintuple bond, depleted uranium, all-metal uranyl, actinide coordination chemistry

## Abstract

While the progression from single (H_2_), double (O_2_) and triple (N_2_) bonds in diatomic molecules to the discovery of transition metal quadruple (Re–Re) and quintuple (Cr–Cr) bonds has significantly expanded our understanding of covalent interactions, the exploration of cumulative multiple bonds in trinuclear systems has remained largely unexplored. Until now, cumulative bonds beyond triple multiplicity have neither been experimentally isolated nor theoretically predicted. Here, we propose a new bonding model—cumulative quintuple bonds—and report the synthesis and characterization of a novel trinuclear species, [Co–U–Co], which exhibits bonding interactions approaching cumulative quintuple bonds. Advanced quantum chemical calculations reveal that the cumulative multiple bonds consist of one pair of U–Co *σ* bonds, two pairs of U–Co *π* bonds, one pair of U–Co *δ* bonds, a three-center-two-electron *δ* bond and a *δ* hyperconjugation. This species represents the first example of an all-metal uranyl analog featuring a linear M–U–M core with cumulative multiple bonds. This discovery not only extends the theoretical boundaries of cumulative multiple bonds but also establishes a transformative precedent for cumulative bonding multiplicity in trinuclear species.

## Teaser

A linear [Co–U–Co] core featuring cumulative multiple bonds is synthesized, extending the long-standing paradigm of bonding multiplicity in trinuclear systems from three to five.

## INTRODUCTION

Chemical bonding is a cornerstone of modern chemistry, providing essential insights into the structure and properties of molecules [1]. Chemists are particularly intrigued by the multiplicity of chemical bonds, as it reflects the multifaceted strength and nature of atomic interactions [2–5]. In the realm of binuclear species, the first three simplest molecules that come to mind are H_2_, O_2_ and N_2_, which feature a single H–H bond, a double O–O bond and a triple N–N bond, respectively (Fig. 1a). For decades, chemists held the belief that the highest multiplicity of chemical bonds was three, as early detected molecules lacked *d* orbitals [6]. This notion was overturned in 1964 when Cotton *et al.* discovered the binuclear complex, [Re_2_Cl_8_]^2−^ [7]. Unlike early molecules, the involvement of Re 5*d* orbitals enabled this complex to exhibit a significant quadruple Re–Re bond, including an extraordinary *δ* bonding interaction. This discovery revolutionized inorganic chemistry, leading to the synthesis of numerous stable molecules with quadruple bonds between transition metals [8–13]. In 2005, the multiplicity of bonds in isolable binuclear complexes reached a new milestone when Power and co-workers reported the complex, [Cr(*μ*-Cl)Ar]_2_ [Ar = C_6_H_3_-2,6(C_6_H_3_-2,6-*^i^*Pr_2_)_2_], featuring a Cr–Cr quintuple bond [14].

**Figure 1.**
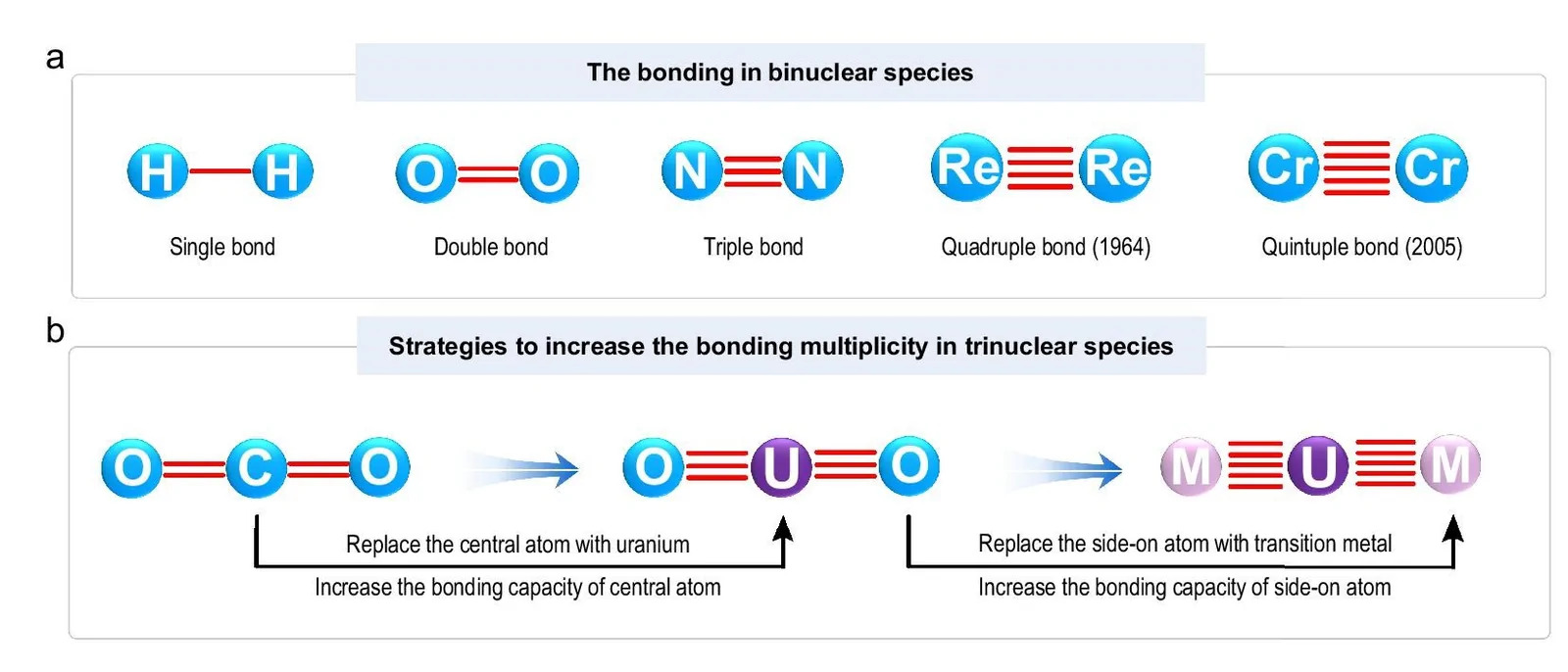
Background and key concepts. (a) Typical bonding in binuclear species. (b) The evolution of cumulative bonds in different species and strategies to increase the bonding multiplicity. By increasing the bonding capacity of the central and side-on atoms, the maximum cumulative bonding multiplicity can approach five. M = transition metal.

However, compared to extensive studies on bonding interactions in binuclear species, research on cumulative multiple bonds in trinuclear species remains limited. One of the most well-known trinuclear compounds with cumulative interactions is carbon dioxide (CO_2_, Fig. 1b). In CO_2_, the carbon center contributes two *sp* and two *p* orbitals, while each oxygen atom supplies one *sp* and one *p* orbital, resulting in cumulative *σ* and *π* bonds. For a long time, it was believed that the maximum multiplicity of cumulative bonds was two, as seen in various allene derivatives [15–18]. Although the carbon center in CO_2_ has already reached the ceiling of its bonding capability, each oxygen atom possesses an additional *p* orbital capable of donating lone pairs. This configuration was realized in the linear trinuclear species, UO_2_^2+^ [19]. Unlike the carbon center, the uranium center exhibits a more diverse bonding capacity due to the participation of *d* and *f* orbitals. The shapes and orientations of these orbitals allow the uranium center to accommodate an extra set of cumulative dative *π* bonds from the oxygen atoms, leading to cumulative triple bonds (Fig. 1b). Since the elucidation of the uranyl cation’s structure, several uranyl derivatives and their isoelectronic species have been synthesized [20–22], none of which surpass a cumulative bonding multiplicity of three. This limitation arises because the side-on atoms in previously isolated linear trinuclear species were restricted to main group elements, which can participate in at most a triple bond, although the possibility of a quadruple bond in C_2_ remains controversial [23–26].

Theoretically, replacing main-group terminal ligands in uranyl with transition metal (M) fragments could generate a cumulative quintuple bond: one pair of *σ* bonds, two pairs of *π* bonds and two pairs of *δ* bonds (Fig. 1b). Here, we propose a new bonding model—cumulative quintuple bonds—which has yet to be theoretically predicted or experimentally observed. In addition, we successfully isolated unprecedented trinuclear clusters featuring a linear [Co–U–Co] core, which exhibit bonding interactions approaching cumulative quintuple bonds. This discovery changes the long-standing dogma that cumulative bonding is capped at a bonding multiplicity of ≤3 (e.g. O=C=O and O≡U≡O), and expands the theoretical boundaries of cumulative multiple bonds.

## RESULTS AND DISCUSSION

### Theoretical model for cumulative quintuple bonds

We begin by establishing a new bonding model: cumulative quintuple bonds in trinuclear systems. Figure 2 illustrates the cumulative quintuple bond orbital interactions in an idealized pure metallic M–U–M cluster, derived from the U *d/f* orbitals and the M *d* orbitals. Specifically, the in-phase and out-of-phase combinations of the ${d}_{{z}^2}$(M) orbitals from the two M centers interact with the ${d}_{{z}^2}$(U) and ${f}_{{z}^3}$(U) orbitals, respectively, forming two 3c–2e *σ* bonds. Additionally, the in-phase and out-of-phase combinations of the ${d}_{xz}$ (Co) orbitals from the two Co centers interact with the ${d}_{xz}( {\mathrm{U}} ){\mathrm{\ and\ }}{f}_{x{z}^2}$(U) orbitals, respectively; likewise, the in-phase and out-of-phase combinations of the ${d}_{yz}$(Co) orbitals from the two Co centers interact with the ${d}_{yz}( {\mathrm{U}} )\ {\mathrm{and\ }}{f}_{y{z}^2}$(U) orbitals, respectively. These four orbital interactions give rise to four 3c–2e *π* bonds. Notably, the versatile bonding capacities of M and U atoms facilitate the formation of four additional 3c–2e *δ* bonds, involving ${d}_{xy}/{d}_{{x}^2 - {y}^2}$ (M) and ${d}_{xy}/{d}_{{x}^2 - {y}^2}/{f}_{xyz}/{f}_{{x}^2z - {y}^2z}$ (U) orbitals. This comprehensive orbital interaction framework supports the cumulative quintuple bonds within the pure metallic M–U–M cluster. To our knowledge, this bonding model has neither been predicted theoretically nor observed experimentally prior to this work.

**Figure 2.**
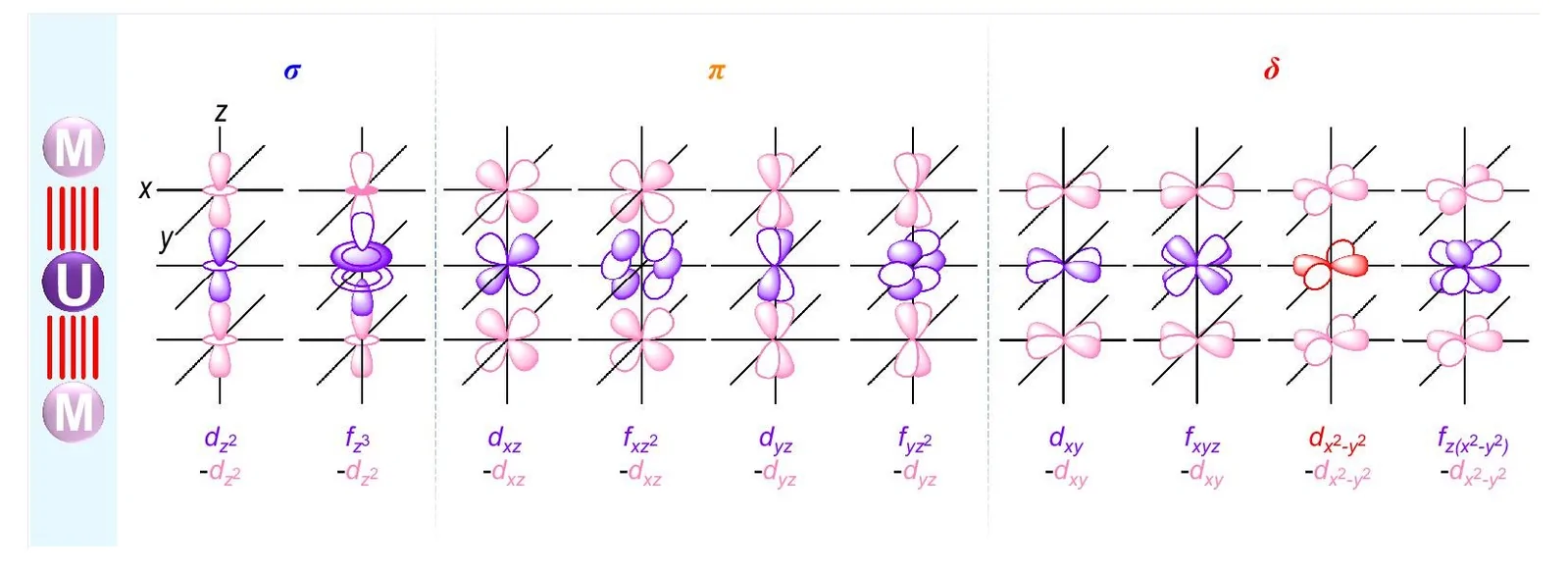
Theoretical model for cumulative quintuple bonds. Bonding orbital interactions of the pure metallic UM_2_ cluster, containing two *σ* bonds, four *π* bonds and four *δ* bonds. M = transition metal.

### Synthesis and characterization

To assemble a molecule with a linear [M–U–M] arrangement, we designed a 1,1′-binaphthyl-based N,P chelating ligand (**L-*S*** or **L-*R***) according to the Hard and Soft Acids and Bases theory. The synthesis commenced with the reaction of uranium tetrachloride (UCl_4_) with two equivalents of **L-*S*** in the presence of *n*-BuLi in tetrahydrofuran (THF) at room temperature (RT), yielding complex **1-*S*** as a red solid in 85% yield. Previous studies have demonstrated that species containing N–P moieties serve as effective precursors for constructing multimetallic clusters with uranium-transition metal bonds [27,28]. Subsequent reaction of **1-*S*** with two equivalents of CoCl_2_ in THF at RT resulted in a color change from orange-red to green, affording **2-*S*** in 82% yield (Fig. 3a). The ^1^H nuclear magnetic resonance (NMR) spectrum of **2-*S*** displayed broad peaks ranging from +56.76 to −44.11 ppm (Fig. S5), consistent with the paramagnetic U(IV) and Co(II) centers. Reduction of **2-*S*** with four equivalents of KC_8_ yielded dark-green crystals of complex **3-*S*** in 35% yield after recrystallization. The moderate isolated yield of **3-*S*** is mainly due to losses during recrystallization; the crude reaction is relatively clean according to NMR. Enantiomers **1-*R*–3-*R*** were synthesized analogously using the **L-*R*** ligand.

**Figure 3.**
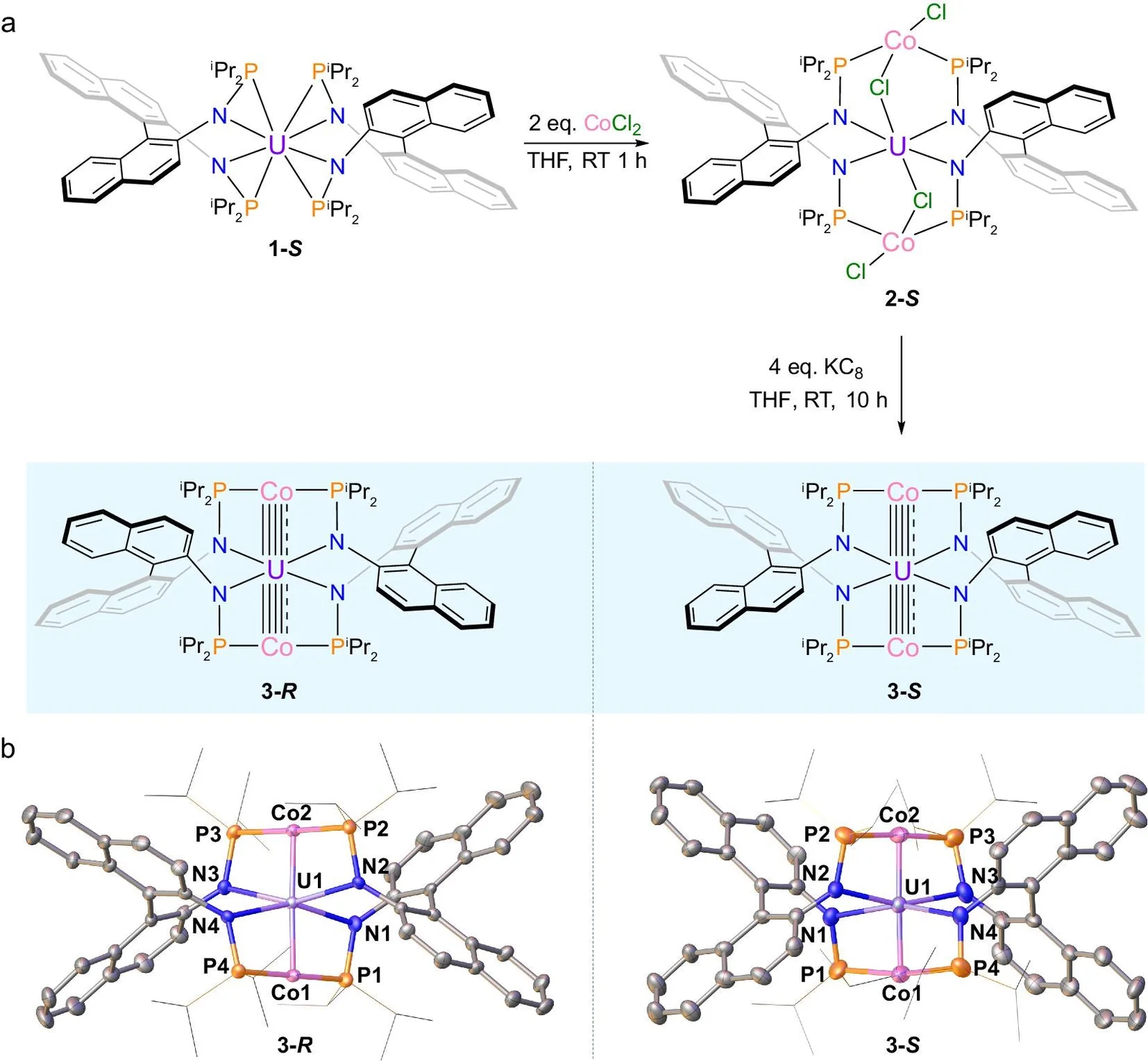
Synthesis and X-ray structure. (a) Schematic representation of the synthetic route to the *S*-configuration tri-metal cluster (**3-*S***) with cumulative multiple bonds. The *R*-configuration enantiomer **3-*R*** was synthesized using analogous procedures with the **L-*R*** ligand. *^i^*Pr, isopropyl; KC_8_, graphitic potassium. (b) Solid-state structures of **3-*R*** and **3-*S***, with *^i^*Pr groups depicted in wireframe and hydrogen atoms omitted for clarity.

Single-crystal X-ray diffraction analysis revealed square-bipyramidal geometry at the hexacoordinate U center in both **3-*S*** and **3-*R***, while the Co centers adopt a T-shaped coordination environment (Fig. 3b). The Flack parameters of 0.068(12) for **3-*S*** and –0.0047(19) for **3-*R*** confirm that the chiral configuration was retained during synthesis. The P–Co–P bond angles are 168.9° and 176.8(2)° in **3-*S*** and 176.42(13)° and 179.46(16)° in **3-*R***. A nearly linear [Co–U–Co] arrangement is evident from the Co–U–Co angles of 175.37(18)° in **3-*S*** and 177.91(7)° in **3-*R***. The U–Co bond lengths in **3-*S*** [2.064(2) and 2.079(2) Å] and in **3-*R*** [2.1260(16) and 2.1215(15) Å] are notably shorter than those reported for U–Co single bonds [2.874(3)–3.0812(7) Å] [29–32], indicating strong U–Co interactions. The formal shortness ratio (FSR)—defined as the observed metal–metal distance divided by the sum of the covalent radii of the two metals—is 0.74 for **3-*S*** and 0.76 for **3-*R***. These values are close to the FSR of 0.75 reported for the Cr–Cr quintuple bond [14]; however, the effective bond order (EBO) per U–Co bond is slightly lower. This is because the Cr–Cr bond is a direct 2c–2e interaction, whereas the U–Co bonds in our system are 3c–2e bonds spanning the Co–U–Co framework, making them more delocalized and susceptible to polarization (*vide infra*). Although the U–Co distances in complexes **3** are comparable to those of known U–Co triple bonds [2.0854(5) and 2.1890(11) Å] [32,33], the lower coordination number of the Co centers in **3** results in a higher bond multiplicity between U and Co (Table S1) [34]. These structural metrics—linear geometry, ultrashort distances and low FSR values—collectively establish complex **3** as the first all-metal structural mimic of the uranyl ion.

### Electronic structure analysis

The ^1^H NMR spectrum of **3-*S*** exhibits sharp resonances in the range of +9.22 to −1.03 ppm, in sharp contrast to the broad, paramagnetically shifted signals of the U(IV) precursor **1-*S*** (Fig. 4a). The absence of large paramagnetic shifts indicates a closed-shell U(VI) (5*f*^0^) and Co(-I) (3*d*^10^) configuration. Consistently, the ^31^P{^1^H} NMR spectrum of **3-*S*** shows a distinct signal at +24.5 ppm (Fig. S7), whereas **1-*S*** and **2-*S*** display no observable ^31^P{^1^H} signals over a wide range (±3000 ppm).

**Figure 4.**
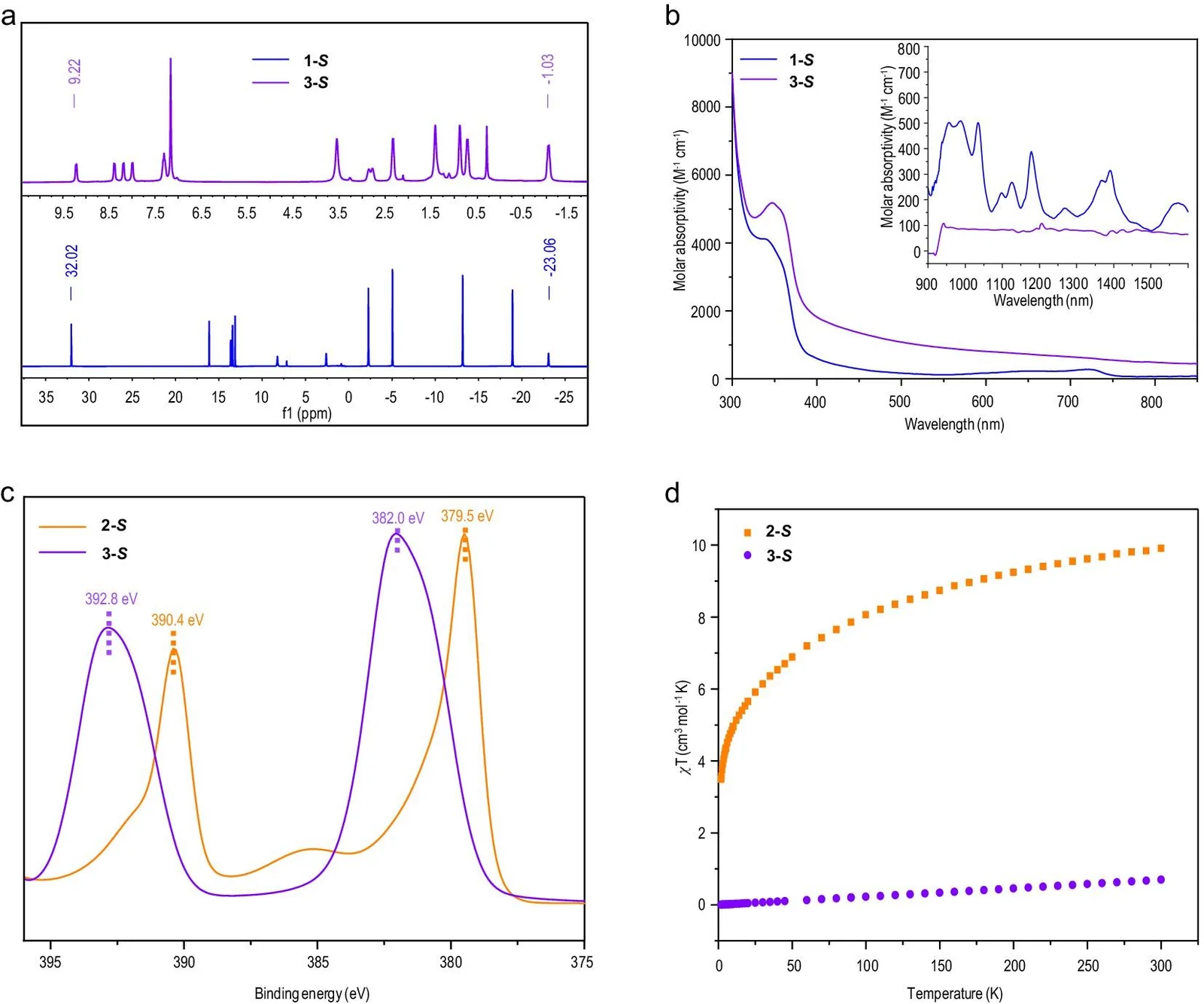
Spectroscopic and magnetic characterization of **3-*S***. (a) The ^1^H NMR spectrum of **3-*S*** displays resonances in the range of +9.22 to −1.03 ppm, which contrasts sharply with the broad, paramagnetically shifted signals (+32.02 to −23.06 ppm) expected for a U(IV) complex **1-*S***. (b) The UV-vis-NIR absorption spectrum of **3-*S*** shows a moderately intense peak at 347 nm but no discernible features in the NIR region. In contrast, the U(IV) reference complex **1-*S*** exhibits characteristic Laporte-forbidden 5*f*→5*f\ *transitions in this region. (c) XPS reveals that the binding energies of U 4*f* in **3-*S*** are significantly higher than those in the U(IV) complex **2-*S***, consistent with a higher oxidation state of uranium in **3-*S***. (d) The magnetic susceptibility (*χ*T) of **3-*S*** is significantly lower than **2-*S*** and approaching 0 at low temperature, which is consistent with the formal oxidation states of U(VI) (5*f*^0^) and Co(-I) (3*d*^10^).

The ultraviolet-visible-near-infrared (UV-vis-NIR) absorption spectrum of **3-*S*** shows a moderately intense peak at 347 nm (*ε* = 5188 M^−1^ cm^−1^) but no discernible features in the near-infrared region (Fig. 4b). The absence of characteristic Laporte-forbidden 5*f*→5*f* transitions is consistent with a 5*f*^0^ electron configuration of U(VI). Time-dependent density-functional theory (TDDFT) calculations predict an absorption peak at 366 nm, attributed to transitions from the highest occupied molecular orbital (HOMO-6 and HOMO-1) to the lowest unoccupied molecular orbital (LUMO+2, LUMO+3 and LUMO+4) (Fig. S35). Notably, some of these transitions can be interpreted as electron transfer from the Co centers to the U center. Excitation at 347 nm results in emission at 425 nm in THF solution at RT (Fig. S16), resembling the emission of hexavalent uranyl species [35].

X-ray photoelectron spectroscopy (XPS) further corroborates the higher oxidation state of uranium in **3-*S*** (Fig. 4c). The binding energies of U-4${f}_{5/2}$ (392.8 eV) and U-4${f}_{7/2}$ (382.0 eV) are significantly higher than those in the U(IV) complex **2-*S*** (390.4 and 379.5 eV, respectively), consistent with a U(VI) assignment [36].

Magnetic susceptibility measurements reveal that the *χ*T product of **3-*S*** is substantially lower than that of **2-*S*** and approaches zero at 1.8 K (Fig. 4d). The nearly temperature-independent paramagnetism observed over a broad temperature range is characteristic of a U(VI) 5*f*^0^ ground state. The small residual susceptibility arises from second-order mixing of low-lying paramagnetic excited states—a Van Vleck-type mechanism previously documented for U(VI)-nitride and U(VI)-acetylide complexes [37,38]. Compared with these typical U(VI) complexes, **3-*S*** exhibits a steeper slope in the *χ*T product, reflecting its more complicated electronic structure, which is dominated by a closed-shell [Co(–I)–U(VI)–Co(–I)] configuration (69%–78%), with minor open-shell contributions (*vide infra*). The energy separations between the ground state and the low-lying paramagnetic excited states were roughly estimated using TDDFT calculations, yielding a value of 0.54 eV (12.5 kcal mol^−1^), indicating a noticeable energetic gap.

The apparent oxidation of uranium from U(IV) in **2-*S*** to U(VI) in **3-*S*** under reducing conditions is unexpected at first glance. However, this seemingly paradoxical outcome arises from an intramolecular redistribution of electron density triggered by reduction of the Co(II) centers. Rather than being localized on uranium, the electrons supplied by KC_8_ are accommodated in molecular orbitals (MOs) that delocalize across the Co–U–Co manifold. This delocalization drives a net shift of electron density away from uranium toward the cobalt centers, resulting in the formal oxidation of uranium to U(VI) alongside reduction of both cobalt atoms to Co(–I).

### Bonding analysis

In the [Co–U–Co]^4+^ metal frameworks of complexes **3**, the dominant electron configuration suggests that the Co atoms adopt a 3*d*^10^ configuration, while the U center exhibits a 5*f*^0^ configuration. As a result, the total of twenty metal valence electrons primarily contribute to the metal–metal interactions within these frameworks. The bonding interactions within the ligand-enclosed [Co–U–Co]^4+^ metal frameworks are more complex than the pure metallic UM_2_ cluster (Fig. 2), requiring detailed computational analyses to delineate the specific metal–metal bonding interactions in complexes **3** (see the Computational details in the Supplementary Information for comprehensive calculations and detailed analysis). Electronic structures were analyzed using the DFT-optimized structure of **3-*S*** (Fig. S32). The average U–Co bond length in this optimized structure is 2.06 Å, closely matching the crystal structure values of 2.064(2) and 2.079(2) Å, thereby validating the computational approach. Additionally, Raman spectroscopy of complex **3-*S*** revealed symmetric and unsymmetric stretches of Co–U–Co at 330 and 404 cm^−1^, respectively (Fig. S18), which align closely with the calculated values of 304 and 379 cm^−1^.

Initial canonical MO analysis revealed extensive mixing of metal–metal interactions with metal–ligand interactions, complicating chemical interpretations (Fig. S33). To probe metal–metal interactions, fragment-aligned molecular orbital (FAMO) analysis was employed [39–41]. By aligning the MOs of the ligand (**L-*S***) and the metal cores (U^6+^ as well as Co^10+^) against the electronic structures of **3-*S***, the non-overlapping parts representing pure metal–metal interactions were identified, yielding a set of FAMOs accommodating the 20 metal valence electrons.

Figure 5 depicts the metal–metal bonding FAMOs within the [Co–U–Co]^4+^ metal framework in the ligated cluster **3-*S***, which are partially comparable to those in the pure metallic UCo_2_^4+^ cluster. The cumulative σ bond interactions are similar to those in Fig. 2, with FAMO1 showing ${d}_{{z}^2}\\!-\\!{d}_{{z}^2}$ interactions and FAMO2 indicating ${f}_{{z}^3}\\!-\\!{d}_{{z}^2}$ interactions. For the cumulative π bonds, although the participating atomic orbitals are similar, the resulting MOs differ slightly due to the more complex and lower symmetric structure of **3-*S***. The four *π* orbitals (FAMO3–FAMO6) are primarily constructed by *df*(U) and *d*(Co) hybrid orbitals. Regarding the cumulative *δ* bonds, the first three *δ* orbitals (FAMO7–FAMO9) consist of *df*(U) and *d*(Co) hybrid orbitals. However, FAMO10 demonstrates that the in-phase combination of the ${d}_{{x}^2 - {y}^2}$ (Co) orbitals interacts less effectively with the remaining orbitals of the U center, indicating a diminished cumulative *δ* bond due to the involvement of the ${d}_{{x}^2 - {y}^2}$ (U) orbital in bonding interactions with the four equatorial N atoms from the ligands.

**Figure 5.**
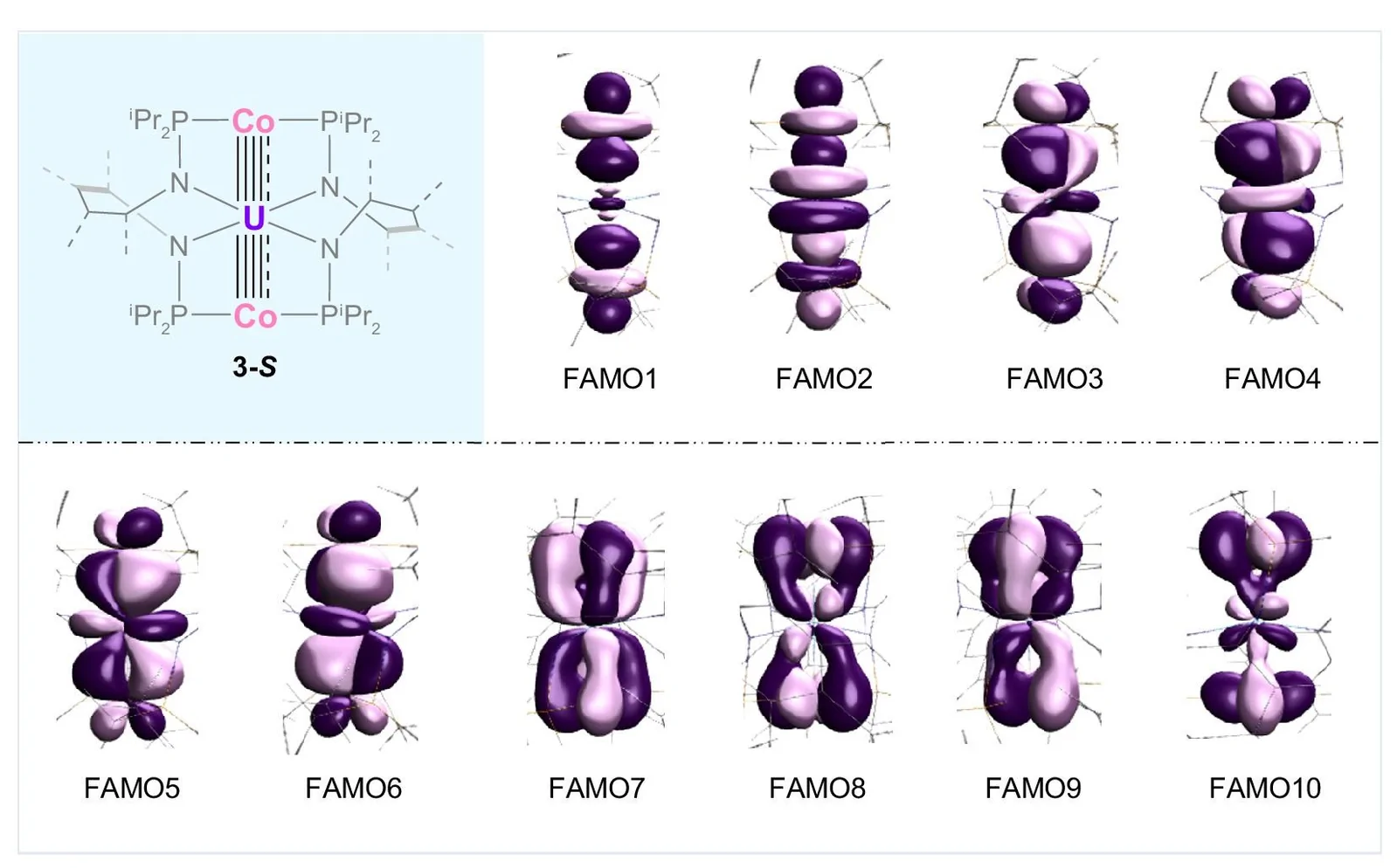
FAMOs analysis. FAMOs (isovalue = 0.150) of the [Co–U–Co]^4+^ metal framework in **3-*S***, classified as *σ* (FAMO1 and FAMO2), *π* (FAMO3 to FAMO6) and *δ* (FAMO7 to FAMO10) types.

Further analysis using the Pipek–Mezey localization method [42] on the FAMOs revealed three types of cumulative bonds: *σ* + *π* + *δ* (Figs S36 and S44). The Hirshfeld method [43–45] indicated that, compared to the electron pairs forming cumulative U–Co *σ* and *π* bonds, those forming U–Co *δ* bonds are more localized on the Co atoms by ∼10%.

To elucidate the participation of the ${d}_{{x}^2 - {y}^2}$ (U) orbital in bonding interactions with the four equatorial N atoms, metal–ligand interactions were analyzed. Figure 6a schematically illustrates the four U–N metal–ligand *σ* bonding interactions, where the linear combinations of the ligand *sp*^2^ (N) hybrid orbitals couple with the $s/{p}_{{\mathrm{hybrid}}}/{d}_{{x}^2 - {y}^2}$ (U) orbitals, forming four N→U *σ* dative bonds. The involvement of the ${d}_{{x}^2 - {y}^2}$ (U) orbital in these N→U bonds disrupts one of the 3c–2e *δ* bonds, resulting in a distinctive *δ* hyperconjugation (Fig. 6b). This preferential orbital engagement originates from the difference in orbital overlap between the two bonding modes. The ${d}_{{x}^2 - {y}^2}$ (U) orbital favors forming strong head-on *σ* overlap with the equatorial nitrogen donors, rather than participating in weak face-to-face *δ* overlap with axial Co centers. Thus, nine 3c–2e metal–metal bonds and one additional *δ* hyperconjugation are identified in the [Co–U–Co]^4+^ framework of **3-*S***.

**Figure 6.**
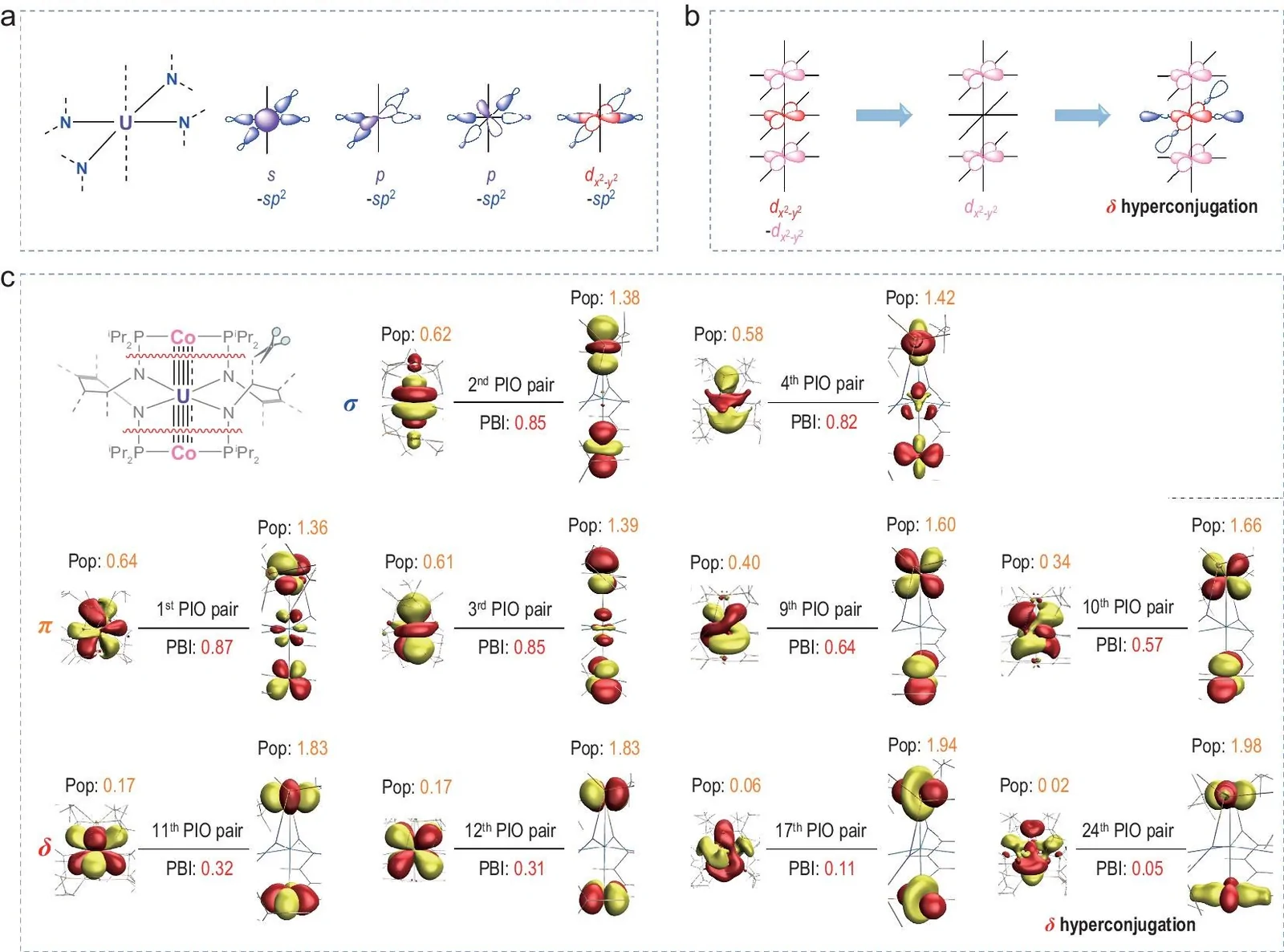
Orbital interactions and principal interacting orbital (PIO) pairs. (a) Bonding orbital interactions between the U center and the ligated N atoms in **3-*S***. (b) Variation from the 3c–2e *δ* bond formed by ${d}_{{x}^2 - {y}^2}$ (U) and ${d}_{{x}^2 - {y}^2}$ (Co) to the *δ* hyperconjugation constructed by the ${d}_{{x}^2 - {y}^2}$ (U)—*sp* (N) anti-bonding orbital and the in-phase combination of ${d}_{{x}^2 - {y}^2}$ (Co). (c) Selected PIO pairs of **3-*S*** (isovalue = 0.250) with corresponding populations (Pops) and PIO-based bond indexes (PBIs). The selected PIO pairs represent the metal–metal interactions and the hyperconjugation, classified as *σ* (top row), *π* (middle row) and *δ* (bottom row) types. The PIO pairs representing the N–P bonding interactions are shown in Fig. S37.

PIO [46,47] analysis was also conducted to quantify the bond orders and electron populations of the interacting orbitals. For **3-*S***, two Co(P*^i^*Pr_2_)_2_ moieties were treated as one fragment, and the remaining part as the other fragment to study the bonding interactions between them (Fig. 6c). The second and fourth PIO pairs denote the cumulative *σ* bond, while the first, third, ninth and tenth PIO pairs represent the cumulative *π* bonds. Additionally, the 11th, 12th and 17th PIO pairs describe the cumulative *δ* bonds, and the 24th PIO pairs indicate the *δ* hyperconjugation.

The average PIO-based bond index (PBI) for the 3c–2e *σ* bonds is 0.84, the mean PBI for the 3c–2e *π* bonds is 0.73 and the average PBI for the 3c–2e *δ* bonds is 0.25. The PBI for the *δ* hyperconjugation is ∼0.05. In the *σ* bonds, the ${d}_{{z}^2}$ (U) and ${f}_{{z}^3}$ (U) orbitals have an average electron population of 0.60, while in the *π* bonds, the ${d}_{xz}$ (U), ${d}_{yz}$ (U), ${f}_{x{z}^2}$ (U) and ${f}_{y{z}^2}$ (U) orbitals have an average population of 0.50. In contrast, the ${d}_{xy}$ (U), ${f}_{xyz}$ (U), ${f}_{{x}^2z - {y}^2z}$ (U) orbitals involved in the *δ* bonding interactions have an average population of 0.13, suggesting that these *δ* bonds are Co→U dative bonds. Focusing on either of the cumulative U–Co bonds, the *σ* bond contributes 0.84, the *π* bonds contribute 1.46 and the *δ* bonds, including *δ* hyperconjugation, contribute 0.40, resulting in a total PBI value of 2.70 (Table S11). It should be noted that the calculated bond order indices are highly sensitive to bond polarity [48]. Although both highly polarized and non-polarized bonds are formally classified as full chemical bonds, polarized bonds consistently yield significantly lower calculated bond orders (Table S11). In the case of **3-*S***, the U–Co bonds are strongly polarized due to their Co→U dative character (Fig. S36), which accounts for the low computed bond order.

### RASSCF analysis

To investigate the plausible multireference character of complex **3-*S*** and to consider the alternative formulations including those antiferromagnetically coupled, RASSCF (restricted active space self-consistent field) analyses were conducted with an optimized simplified model, **3′**. In **3′**, all substituents on P and N are converted to methyl groups (Fig. S38). The U–Co, U–N, Co–P and P–N bond lengths in **3** and **3′** are comparable (Tables S10 and S12), rendering **3′** as an ideal simplified model. The truncated model is applied to keep the calculations tractable with the resources available.

For RASSCF calculations, RAS(20,1,1; 4,12,4) active space is first applied [49]. As shown in Fig. 7, RAS2 contains two *σ* bonding orbitals, two *σ* anti-bonding orbitals, four *π* bonding orbitals and four *π* anti-bonding orbitals. Besides, RAS1 includes four *δ* type orbitals, while RAS3 contains four *δ* anti-bonding orbitals. Notably, active orbital 2 is indeed a non-bonding *δ* orbital, additionally verifying the partial diminishment of the fifth cumulative bond. This fact is supported by our bonding scheme analysis as well as the MO interaction diagram for the RASSCF calculations (Figs S41 and S42). The occupation numbers of the *δ* bonding orbitals are slightly larger than those of the *σ* and *π* bonding orbitals, inherently due to the relatively higher polarity of the *δ* bonds compared to the *σ* and *π* bonds (Fig. S36).

**Figure 7.**
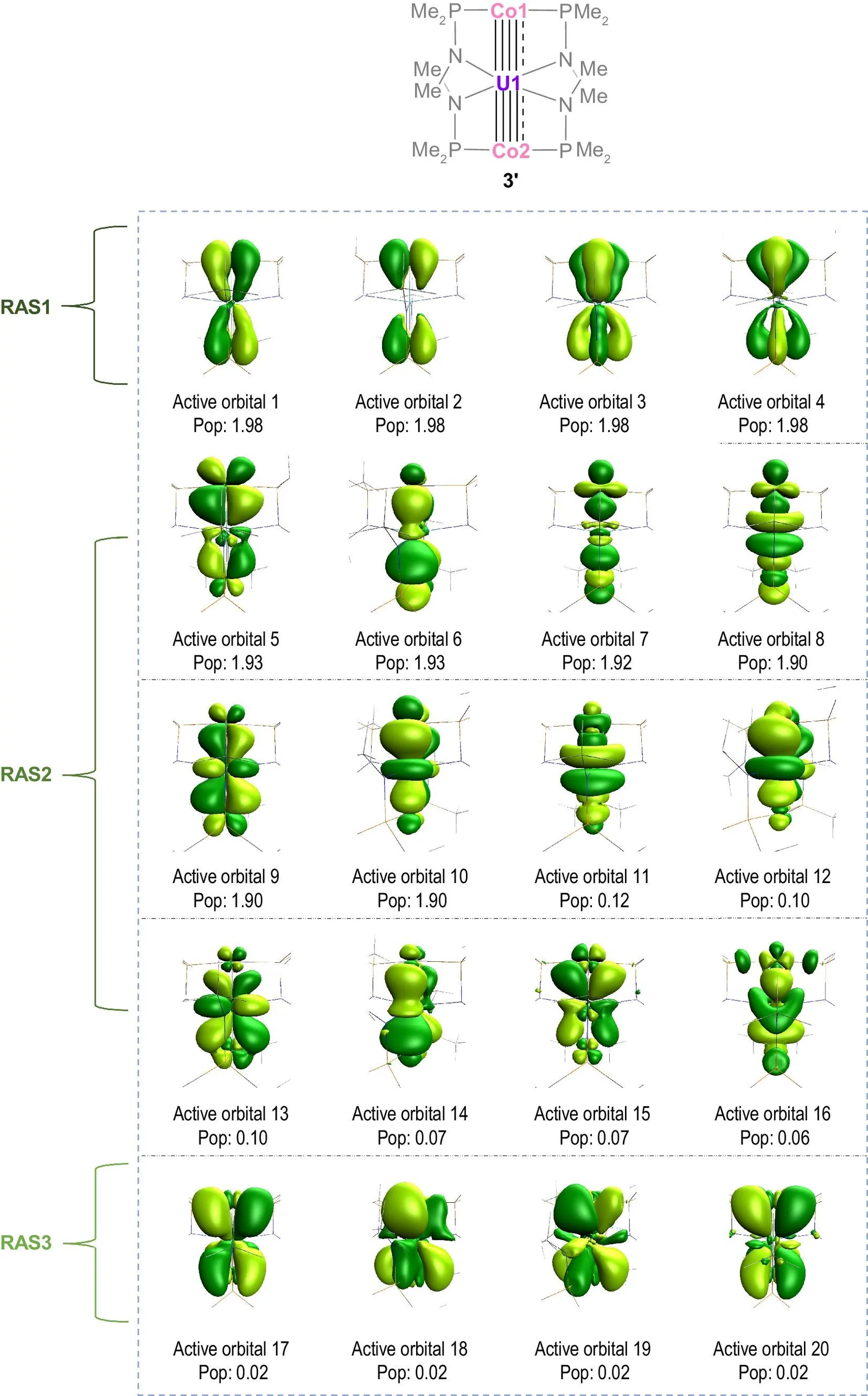
RASSCF results for **3′**. Active orbitals (isovalue = 0.150) are shown with their corresponding occupation numbers (rounded to two decimal places), as obtained from the RASSCF calculation. The active space is partitioned into three subspaces: RAS1, RAS2 and RAS3. In RAS1, four orbitals are constrained to be doubly occupied, allowing a maximum of one hole. RAS2 comprises twelve orbitals with twelve electrons, where all possible occupations are permitted. In RAS3, four orbitals are formally empty, with a maximum of one electron allowed. The complete active space can be denoted as RAS(20,1,1; 4,12,4). For orbital plots at a higher isovalue, refer to Fig. S40. MO interaction diagrams illustrating these orbitals are provided in Figs S41 and S42.

The electron configuration composition (Table S14) indicates that the dominant electron configuration contributes 69.5%. For **3′**, the dominant electron configuration is closed-shell, resulting in a 3*d*^10^ configuration for both Co(−I) centers, which may give rise to the double-shell effect [50]. Besides, the other electron configurations with proportions >1.0% exhibit antiferromagnetic coupling.

To further validate the bonding multiplicity of complexes **3**, the EBO [4] was quantified. The EBO values are calculated using the following equation, EBO = (number of bonding electrons − number of anti-bonding electrons)/2. Based on the RASSCF results, the maximum EBO for the cumulative U–Co bonds in **3′** is 4.21, confirming that the multiplicity of the U–Co bonds in complexes **3** is beyond triple. Furthermore, although the existence of *δ* bonds has already been confirmed, the minimum EBO for the U–Co bonds would be 2.74, if we only consider the *σ* and *π* bonds. By comparing the EBO (2.74–4.21) to the ideal bonding multiplicity (3.0–4.5), the percentage of radical character in **3′** was analyzed, spanning from 6.4% to 8.7% [51]. Notably, this modest radical character is of predominantly static multireference origin. It arises from the mixing of low-lying open-shell electronic configurations into the dominant closed-shell state (Table S14). The near-degeneracy between these electronic states gives rise to the intrinsic multireference nature of the system.

## CONCLUSION

Over a century ago, Gilbert Lewis introduced the foundational model of covalent bonding, emphasizing electron pairing between neighboring atoms [1]. The enduring question of how many bonds can form between two atoms has driven decades of investigation. Groundbreaking works by Cotton [7] and Power [14] on isolating binuclear species with quadruple and quintuple bonds have significantly advanced our understanding of chemical bonding. Yet, until now, cumulative triple bonds remained the highest bonding multiplicity observed in trinuclear molecules. In this study, we proposed a new bonding motif—cumulative quintuple bonds—which, to our knowledge, had neither been experimentally observed nor theoretically predicted. Although the idealized model formally supports a cumulative quintuple bond, the actual complex with [Co–U–Co] unit shows a slightly lower EBO because of polarization and ligand competition. Rather than serving as mere structural scaffolding, ligand environment actively modulates the metal–metal bonding multiplicity. Specifically, the introduction of either the **L-*S*** or **L-*R*** ligand converts one of the 3c–2e metal–metal *δ* bonds into *δ* hyperconjugation, as one of the U *d* orbitals is diverted to participate in metal–ligand interactions. Such linear trimetallic chains featuring metal–metal multiply bonded motifs have emerged as promising competitors for catalytic and molecular electronics applications [52]. Unlike previously reported linear M1–M2–M2 systems [52], which feature localized M2–M2 multiple bonds, our [Co–U–Co] architecture adopts an M1–M2–M1 arrangement with cumulative bonds from the central U to both terminal Co centers.

Moreover, this work delivers the long-sought all-metal member of the uranyl analog family, expanding the [E≡U≡E]*^n^*^+^ paradigm from main-group elements to transition metals. The [Co–U–Co]^4+^ core provides a molecular platform for probing U–M multiple bonding at the discrete-molecule level—a regime with direct relevance to uranium-based superconducting intermetallics such as UGe_2_ [53], URhGe [54], UCoGe [55] and UAs_2_ [56]. The design principles established here should enable a broader class of [M–U–M]*^n^*^+^ systems, such as putative [Fe–U–Fe] and other first-row analogues, opening a new avenue at the interface of molecular uranium chemistry and condensed-matter physics.

## METHODS

Experiments were performed under an Ar or N_2_ atmosphere using standard Schlenk-line or glove-box techniques. Solvents were dried and degassed with a solvent purification system before use. See the Supplementary Information for detailed experimental procedures, crystallographic and computational details.

## Supplementary Material

nwag462_Supplemental_Files

## Data Availability

The X-ray crystallographic coordinates for structures reported in this article have been deposited at the Cambridge Crystallographic Data Centre (CCDC), under deposition nos. CCDC-2388408 (**1-*R***), 2388409 (**1-*S***), 2388410 (**2-*R***), 2388411 (**3-*R***) and 2388412 (**3-*S***). These CCDC data can be obtained free of charge from the CCDC *via*  https://www.ccdc.cam.ac.uk/structures/. The data that support the findings of this study are available from the corresponding author upon request.
