# Supplementary material for: Approaching cumulative quintuple bonds in all-metal uranyl analogs
Source: Natl Sci Rev. 2026 Jul 30;13(18):nwag462. doi: 10.1093/nsr/nwag462 (PMC13637622; doi:10.1093/nsr/nwag462)
Supplement: Supplementary file 1 [file nwag462_supplemental_files.zip › Supplementary_Information_NSR-2026-1960.pdf]

Supplementary Information for

Approaching Cumulative Quintuple Bonds in All-Metal Uranyl Analogs

Xingxing Gong,[1†] Jiaxin Chen,[2†] Xinyu Liu,[1†] Ningxin Jiang,[1] Jingxiang Zou,[4] Yichi Zhang,[2] Yue Zhao,[1] Shuao Wang,[3] Zhenyang Lin,[2]* and Congqing Zhu[1]*

Affiliations:

[1] State Key Laboratory of Coordination Chemistry, Jiangsu Key Laboratory of Advanced Organic Materials, School of Chemistry and Chemical Engineering, Nanjing University, Nanjing 210023, China

[2] Department of Chemistry, The Hong Kong University of Science and Technology, Clearwater Bay, Kowloon, Hong Kong, 999077, China

[3] State Key Laboratory of Radiation Medicine and Protection, School for Radiological and interdisciplinary Sciences (RAD-X) and Collaborative Innovation Center of Radiation Medicine of Jiangsu Higher Education Institutions, Soochow University, Suzhou 215123, China

[4] Laboratory of AI for Electrochemistry (AI4EC Lab), Tan Kah Kee Innovation Laboratory (IKKEM), Xiamen 361005, China.

[†]These authors contributed equally to this work.

* Correspondence and requests for materials should be addressed to Z.L. (chzlin@ust.hk) or C.Z. (E-mail: zcq@nju.edu.cn).

Contents

1. Experimental Procedure……………………………………………………S1

2. Supplementary Figures…...…………………………………………………......S5

3. X-ray Diffraction Data…………..……………………………..…………S18

4. Computational details…………………………………………..…………S25

5. References………………………………………………………………S50

1. Experimental Procedure

General Procedure: All manipulations were performed under N_2 atmosphere using standard Schlenk techniques or in a glovebox. Commercially available chemicals were used as received without further purification. The solvents were obtained by passing through a solvent purification system and further dried over 4 Å molecular sieves. Benzene-d_6 and THF-d_8 were dried over Na/K and stored under a N_2 atmosphere prior to use. Nuclear magnetic resonance spectroscopy was performed using a Bruker AVIII-400 (^{1}H 400 MHz; ^{13}C{^{1}H} 101 MHz; ^{31}P{^{1}H} 162 MHz) spectrometer at room temperature (RT). The ^{1}H and ^{13}C{^{1}H} NMR chemical shifts (δ) are relative to tetramethylsilane, and ^{31}P{^{1}H} NMR chemical shifts are relative to 85% H_3PO_4. Absolute values of the coupling constants are provided in Hertz (Hz). Multiplicities are abbreviated as singlet (s), doublet (d), triplet (t), multiplet (m), and broad (br). Magnetic measurements were performed on crystalline samples using a Quantum Design SQUID magnetometer at temperatures ranging from 1.8 K to 300 K. The sample was added to a pre-weighed SQUID capsule in a glovebox. The capsule was then sealed, weighed, and transferred to the SQUID cavity for the magnetic measurement. All magnetic data were corrected for the diamagnetic contributions of the sample holder and of the core diamagnetism of the samples using Pascal's constant. Elemental analyses (C, H, N) were performed on a Vario MICRO elemental analyser at the Center of Modern Analysis of Nanjing University. Fourier transform infrared (FT-IR) spectra were performed on Bruker-VERTEX80V. High Resolution Mass Spectra were obtained from on an Agilent 6540 Q-TOF mass spectrometer, operating electrospray ionization (ESI) mode. Fluorescence quenching studies were performed using a Hitachi F-7000 Fluorescence Spectrophotometer. Raman spectroscopy was tested on HORIBA HR Evolution Raman microscope with laser wavelength of 1064 nm. XPS measurements were carried out at 298 K on a Thermo Fisher K-Alpha X-ray Photoelectron Spectrometer. Correction of the charge effect was made with the C1s peak at 284.8 eV. Peak decomposition was performed using the Avantage software of Thermo. Circular dichroism (CD) spectra were performed on a JASCO J-810 spectropolarimeter with 200 nm/min of scan speed.

Synthesis of L-*S*

A solution of n-BuLi (8 mL, 2.5 M in hexane, 20.0 mmol, 2 equiv.) was added dropwise to a solution of (*S*)-(-)-2,2'-diamino-1,1'-binaphthalene (2.84 g, 10.0 mmol, 1 equiv.) in THF (20 mL). The mixture was stirred for 1 hour at room temperature (RT), after which $^{i}Pr_2PCl$ (20.0 mmol, 2 equiv.) was added dropwise. The mixture was stirred for an additional 3 hours at RT and then filtered through celite. The solvents were removed under reduced pressure. The yellow oil was extracted with hexane and filtered through diatomite. The solvents were again removed under reduced pressure, yielding compound **L-*S*** as a colorless oil (3.87 g, 75%). The ligand **L-*R*** (4.02 g, 78%) was synthesized using (*R*)-(-)-2,2'-diamino-1,1'-binaphthalene following the same procedure. **L-*R*** and **L-*S*** show identical results in nuclear magnetic resonance spectroscopy, UV-vis-NIR spectrum, and FT-IR measurements. ^{1}H NMR (400 MHz, Benzene-d_6) δ ^{1}H NMR (400 MHz, Benzene-d_6) δ 8.30 (dd, *J* = 9.0, 4.8 Hz, 2H), 7.76 (d, *J* = 9.0 Hz, 2H), 7.67 (d, *J* = 7.8 Hz, 2H), 7.21 (d, *J* = 8.4 Hz, 2H), 7.13 – 7.09 (m, 2H), 7.06 – 7.01 (m, 2H), 4.08 (d, *J* = 10.8 Hz, 2H), 1.32 – 1.26 (m, 2H), 1.13 – 1.09 (m, 2H), 0.93 – 0.85 (m, 12H), 0.76 (dd, *J* = 16.2, 7.1 Hz, 6H), 0.49 (dd, *J* = 10.2, 6.9 Hz, 6H). ^{13}C NMR (101 MHz, Benzene-d_6) δ 145.79, 145.63, 133.86, 129.52, 129.50, 128.57, 128.28, 126.82, 124.44, 122.54, 117.24, 117.00, 113.65, 113.63, 26.39, 26.36, 26.28, 26.23, 18.50, 18.30, 17.28, 17.18, 15.78, 15.71. ^{31}P NMR (162 MHz, Benzene-d_6) δ 45.7. FT-IR ν/cm^{-1} (KBr): 3477, 3382, 3342, 3048, 2951, 2863, 1614, 1505, 1465, 1398, 1341, 1281, 1239, 1145, 982, 807, 749. HRMS (ESI) calcd. for $C_{32}H_{42}N_2P_2Na$ $[M+Na]^+$ m/z 539.2715, found 539.2720.

Synthesis of complex 1-*S*

A 2.5 M solution of n-BuLi in hexane (0.8 mL, 2.0 mmol, 2 equiv.) was added dropwise to a solution of **L-*S*** (516 mg, 1.0 mmol, 1 equiv.) in THF (10 mL) at -30 °C. The mixture was allowed to warm to RT and stirred for an additional hour. Subsequently, a solution of UCl_4 (190 mg, 0.5 mmol, 0.5 equiv.) in THF (2 mL) was added. The mixture was stirred overnight at RT, and the solvents were then removed under reduced pressure. The residues were extracted with hexane. After being stored at RT for one day, red

crystals of complex **1-*S*** suitable for X-ray diffraction were obtained. Yield: 538.6 mg, 85%. The enantiomer **1-*R*** (500.5 mg, 79%) was synthesized using the same procedure by the reacting **L-*R*** and UCl_4 in the presence of n-BuLi. **1-*R*** and **1-*S*** exhibit identical data in nuclear magnetic resonance spectroscopy, elemental analysis, UV-vis-NIR spectrum, FT-IR, and SQUID measurements. ^{1}H NMR (400 MHz, Benzene-d_6) δ 32.03 (d, J = 8.0 Hz, 4H), 16.11 (t, J = 7.8 Hz, 4H), 13.63 (d, J = 6.6 Hz, 4H), 13.42 (d, J = 7.5 Hz, 4H), 13.12 (t, J = 7.7 Hz, 4H), 8.23 (s, 4H), 2.60 (s, 4H), -2.28 (d, J = 6.0 Hz, 12H), -5.06 (d, J = 5.7 Hz, 12H), -13.16 (d, J = 6.5 Hz, 12H), -18.92 (d, J = 6.5 Hz, 12H), -23.08 (s, 4H). The ^{31}P NMR spectrum of **1** is silent in the range of +3000 to -3000 ppm, which is probably due to the presence of paramagnetic U(IV) center. Anal. Calcd for $C_{64}H_{80}N_4P_4U$: C, 60.66; H, 6.36; N, 4.42; Found: C, 60.86; H, 6.40; N, 4.38. FT-IR ν/cm^{-1} (KBr): 3242, 3119, 3012, 2780, 2695, 1780, 1649, 1531, 1485, 1428, 1374, 1298, 1175, 1112, 1052, 952, 902, 843, 772, 723.

Synthesis of complex 2-*S*

A tetrahydrofuran solution (1 mL) of $CoCl_2$ (51.9 mg, 0.4 mmol, 2 equiv.) was added to a stirred solution of complex **1-*S*** (253.5 mg, 0.2 mmol, 1 equiv.) in THF (10 mL) at RT. The mixture was stirred at RT for 1 hour, during which the color of the solution changed from orange-red to dark green. The solution was then filtered and concentrated to 5 mL, and stored at ambient temperature overnight to yield crystalline powder. Washing the powder with cold THF afforded pure **2-*S***. Yield: 250.4 mg, 82%. The enantiomer **2-*R*** (241.3 mg, 79%) was synthesized using the same procedure by the reacting **1-*R*** and $CoCl_2$ in THF at RT. **2-*R*** and **2-*S*** exhibit identical data in nuclear magnetic resonance spectroscopy, elemental analysis, UV-vis-NIR spectrum, FT-IR, and SQUID measurements. ^{1}H NMR (400 MHz, THF-d_8, 298 K) δ: 56.76 (br), 54.30 (br), 32.19 (br), 30.76 (br), 30.30 (br), 24.25 (br), 17.56 (br), 16.67 (br), 14.47 (br), 14.27 (br), 13.84 (br), 11.84 (br), -15.07 (br), -33.58 (br), -44.11 (br). The ^{31}P NMR spectrum of **2** is silent in the range of +3000 to -3000 ppm, which is probably due to the presence of paramagnetic U(IV) and Co(II) ions. Anal. Calcd for $C_{46}H_{38}Cl_4Co_2N_4P_4U$: C, 50.34; H, 5.28; N, 3.67; Found: C, 50.09; H, 5.36; N, 3.56. FT-

IR ν/cm^{-1} (KBr): 3055, 2961, 2925, 2870, 2376, 1589, 1501, 1458, 1394, 1308, 1255, 1196, 1143, 1098, 1069, 1026, 989, 955, 834, 803, 727, 669.

Synthesis of complex 3-*S*

A suspension of KC_8 (32.4 mg, 0.24 mmol, 4 equiv.) in THF (2 mL) was added to a solution of complex **2-*S*** (91.6 mg, 0.06 mmol) in THF (2 mL). The mixture was stirred at RT for 10 h. The mixture was then filtered and the solvents were removed under reduced pressure to give complex **3-*S*** as a black solid. Dark green crystals (24.9 mg, 30%) were successfully grown from a saturated solution stored at RT. The enantiomer **3-*R*** (29.1 mg, 35%) was synthesized using the same procedure by reacting **2-*R*** and KC_8 in THF at RT. **3-*R*** and **3-*S*** exhibit identical data in nuclear magnetic resonance spectroscopy, elemental analysis, UV-vis-NIR spectrum, and FT-IR measurements. ^{1}H NMR (400 MHz, Benzene-d_6, 298 K) δ 9.21 (d, Δν½ = 17.1 Hz, 4H), 8.38 (d, Δν½ = 17.4 Hz, 4H), 8.19 (d, Δν½ = 17.6 Hz, 4H), 7.99 (d, Δν½ = 17.1 Hz, 4H), 7.34 – 7.26 (m, Δν½ = 29.9 Hz, 8H), 2.89 – 2.78 (m, Δν½ = 62.5 Hz, 8H), 2.36 – 2.30 (m, Δν½ = 23.5 Hz, 12H), 0.90 – 0.86 (m, Δν½ = 21.1 Hz, 12H), 0.75 – 0.68 (m, Δν½ = 27.7 Hz, 12H), -1.01 – -1.08 (m, Δν½ = 30.0 Hz, 12H). ^{31}P NMR (162 MHz, Benzene-d_6) δ 24.5. Anal. Calcd for $C_{64}H_{80}Co_2N_4P_4U$: C, 55.50; H, 5.82; N, 4.04; Found: C, 55.56; H, 5.91; N, 3.98. FT-IR ν/cm^{-1} (KBr): 3049, 2956, 2921, 2863, 1901, 1612, 1589, 1504, 1461, 1396, 1335, 1262, 1095, 1026, 873, 803, 742. μ_{eff} (Evans method, Benzene-d_6, 298 K): 1.65 μ_B.

2. Supplementary Figures

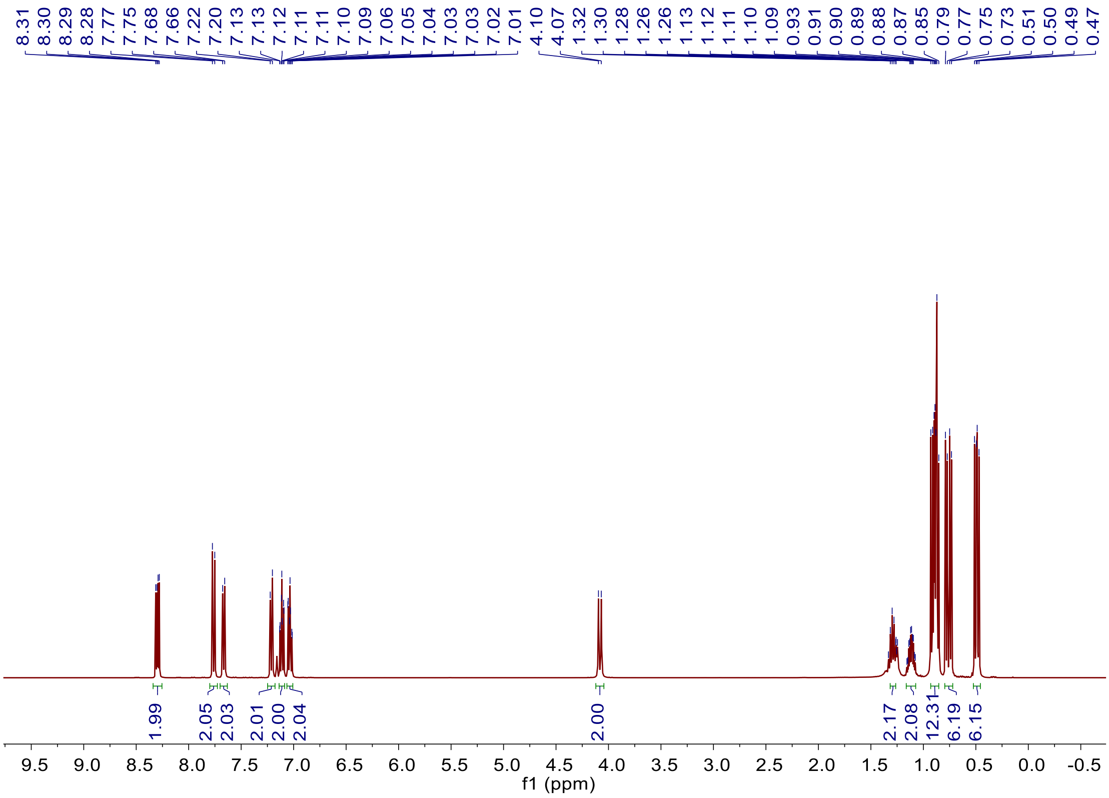

Figure S1. The ^{1}H NMR (400 MHz) spectrum of ligand **L-*S*** in C_6D_6.

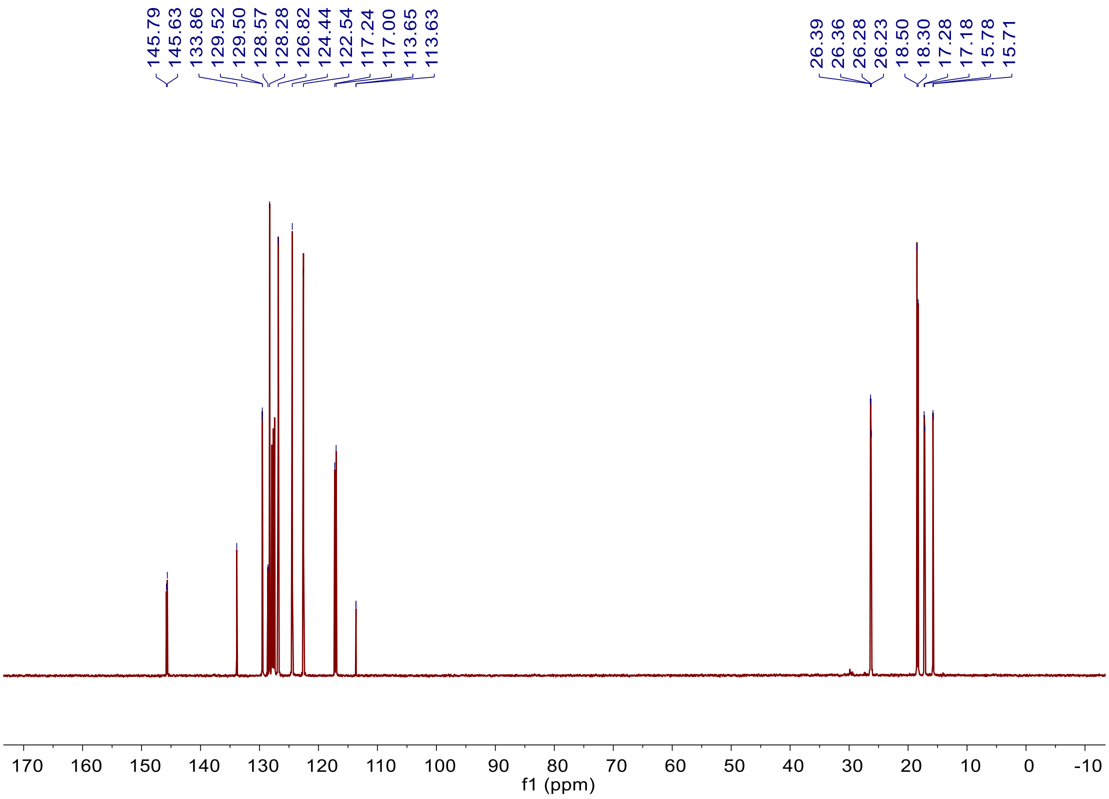

Figure S2. The ^{13}C NMR (101 MHz) spectrum of ligand **L-*S*** in C_6D_6.

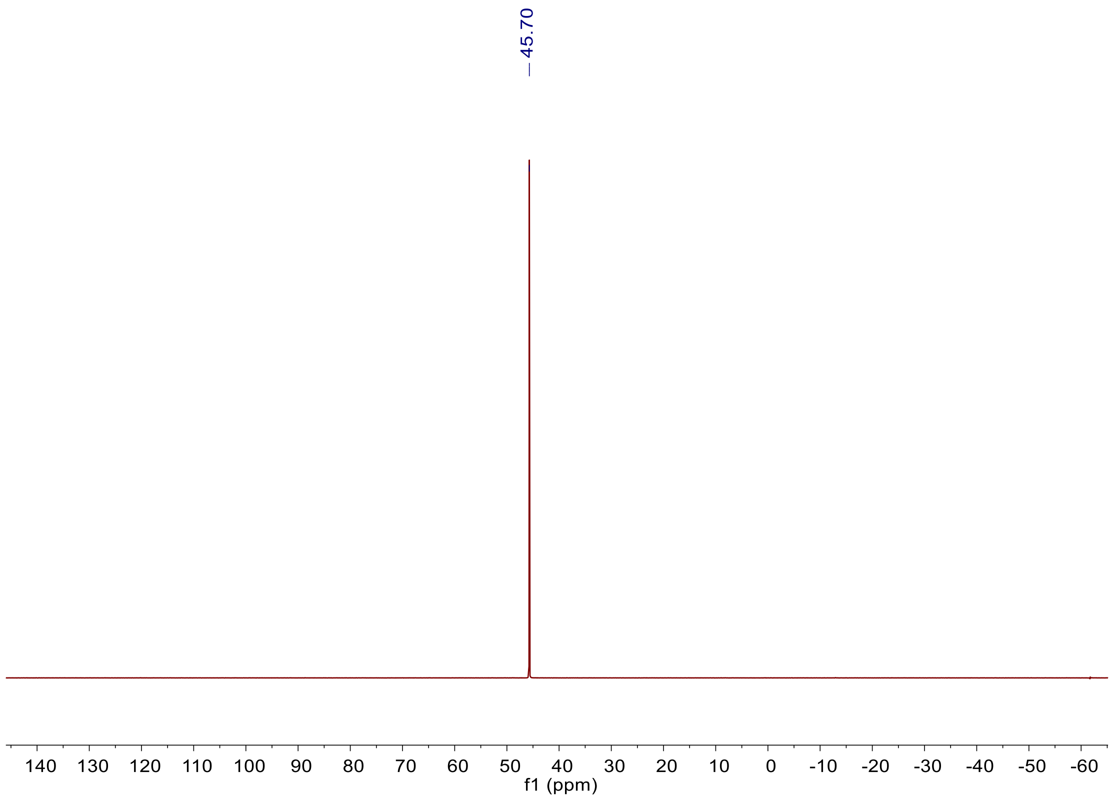

Figure S3. The $^{31}P\{^{1}H\}$ NMR (162 MHz) spectrum of ligand **L-*S*** in C_6D_6.

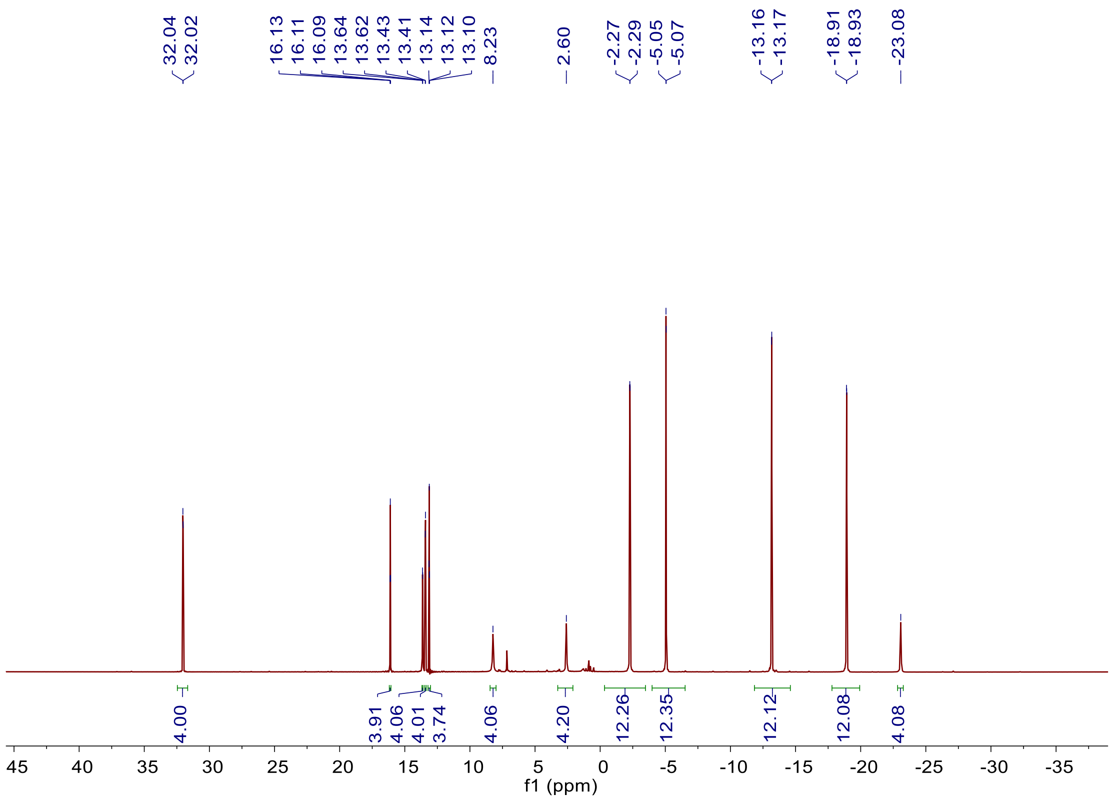

Figure S4. The ^{1}H NMR (400 MHz) spectrum of complex **1-*S*** in C_6D_6.

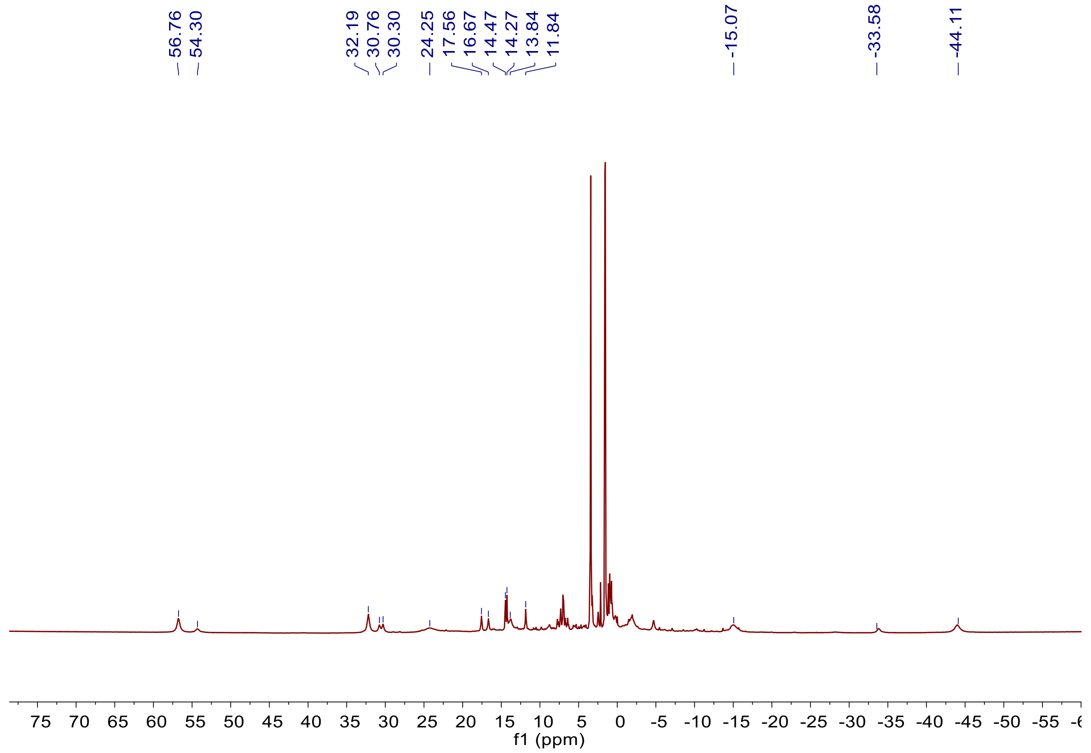

Figure S5. The ^{1}H NMR (400 MHz) spectrum of complex **2-*S*** in THF-d_8. Due to the poor solubility of **2-*S*** in THF-d_8 after recrystallization, the spectrum quality is not perfect. However, the signals ranging from +56.76 to -44.11 ppm indicate that this complex is paramagnetic, potentially containing both U(IV) and Co(II) centers.

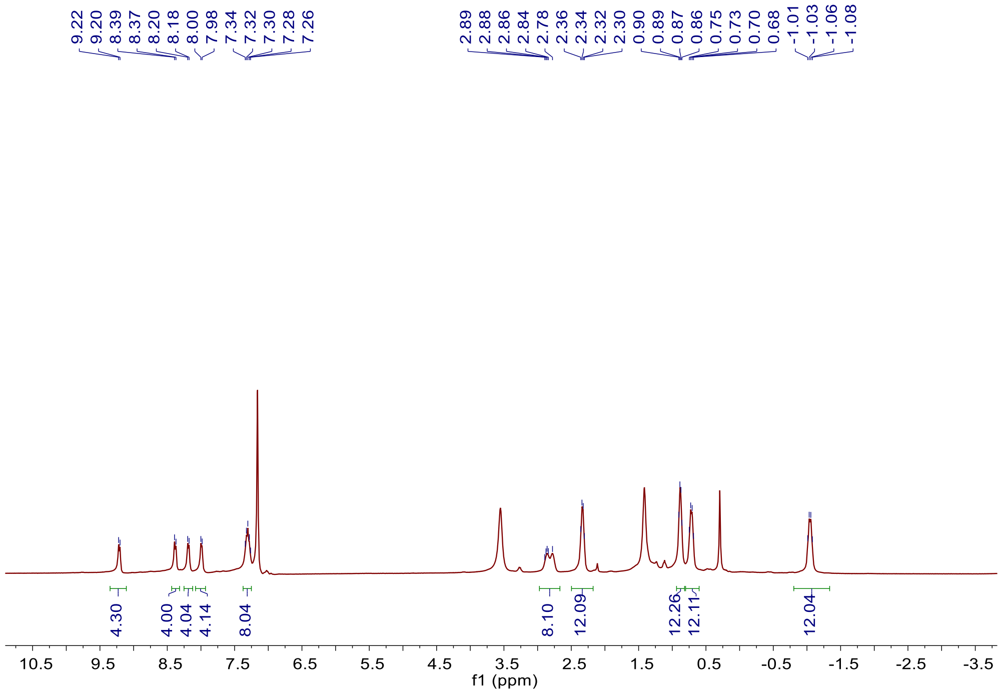

Figure S6. The ^{1}H NMR (400 MHz) spectrum of complex **3-*S*** in C_6D_6.

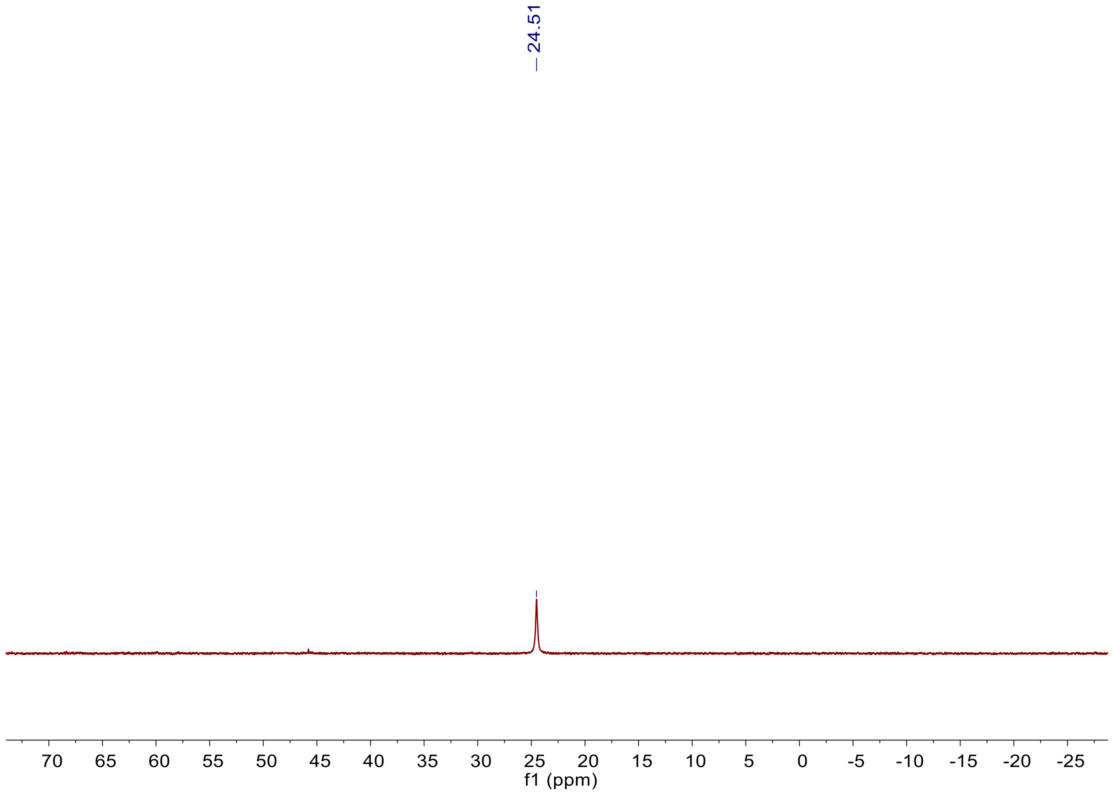

Figure S7. The $^{31}P\{^{1}H\}$ NMR (162 MHz) spectrum of complex **3-*S*** in C_6D_6.

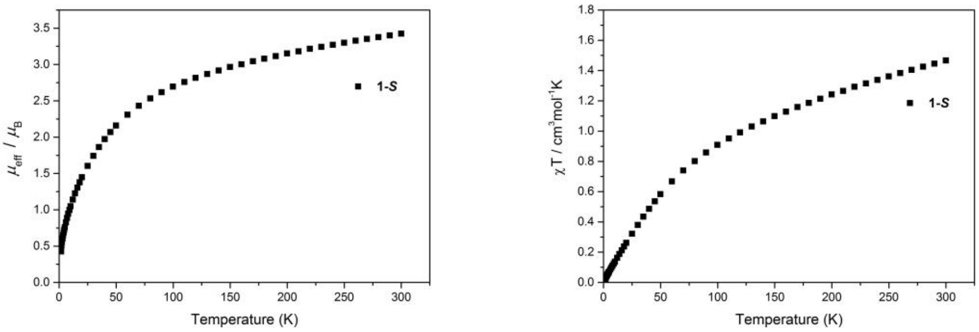

Figure S8. Variable-Temperature SQUID magnetism of **1-*S*** (μ_{eff} vs T and χT vs T).

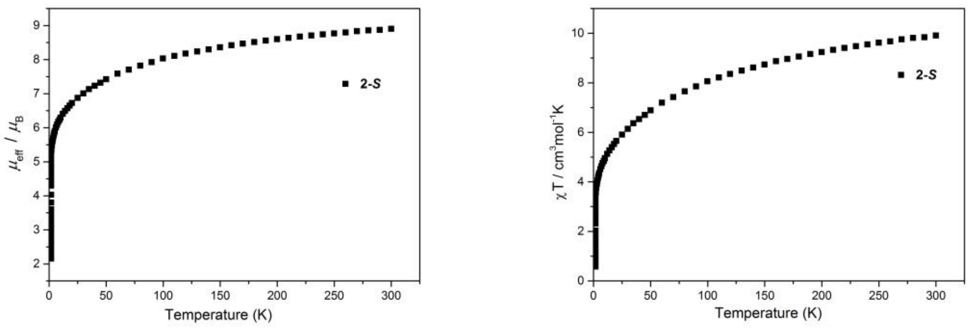

Figure S9. Variable-Temperature SQUID magnetism of **2-*S*** (μ_{eff} vs T and χT vs T).

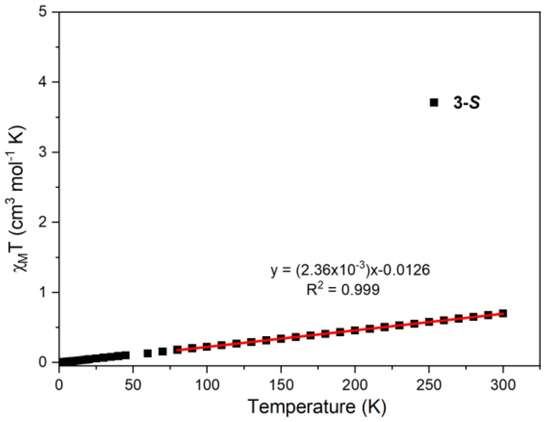

Figure S10. Magnetic susceptibility χT vs T for **3-*S*** over the temperature range 2-300 K. Linear regression over the range 85-300 K gives a TIP of 2.36 × 10^{-3} cm^3 mol^{-1} K with R^2 = 0.999.

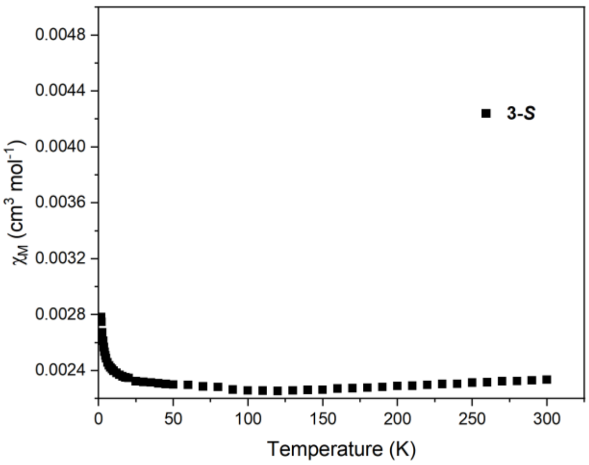

Figure S11. Magnetic susceptibility χ vs T for **3-*S*** over the temperature range 2-300 K.

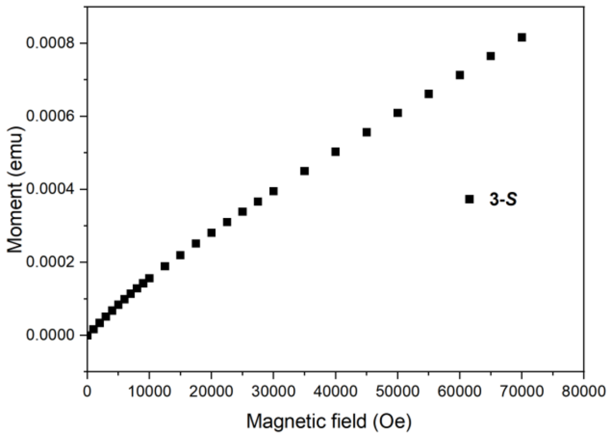

Figure S12. The field-dependent magnetization (M vs H) data for **3-*S***.

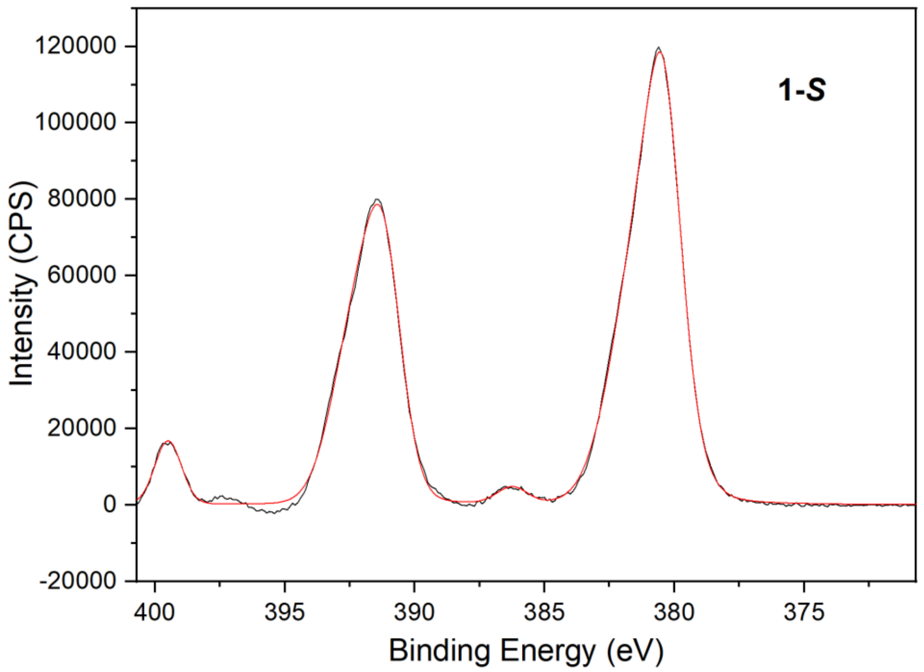

Figure S13. The Shirley background-subtracted XPS spectrum (black) and the fitting curve (red) of **1-*S***. Binding energies: U $4f_{5/2}$, 391.0 eV; U $4f_{7/2}$, 380.1 eV.

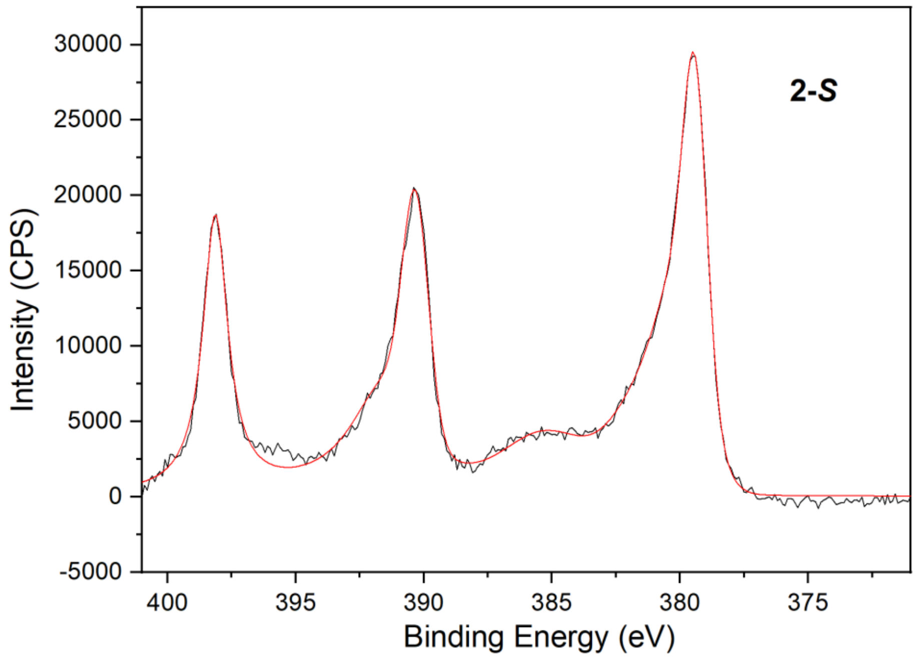

Figure S14. The Shirley background-subtracted XPS spectrum (black) and the fitting curve (red) of **2-*S***. Binding energies: U $4f_{5/2}$, 390.4 eV; U $4f_{7/2}$, 379.5 eV.

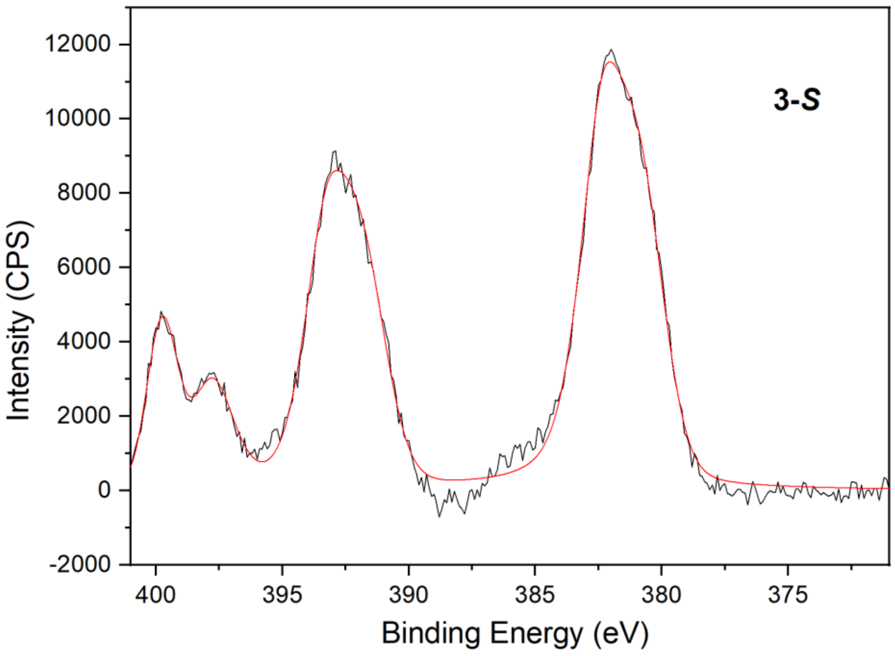

Figure S15. The Shirley background-subtracted XPS spectrum (black) and the fitting curve (red) of **3-*S***. Binding energies: U 4$f_{5/2}$, 392.8 eV; U 4$f_{7/2}$, 382.0 eV.

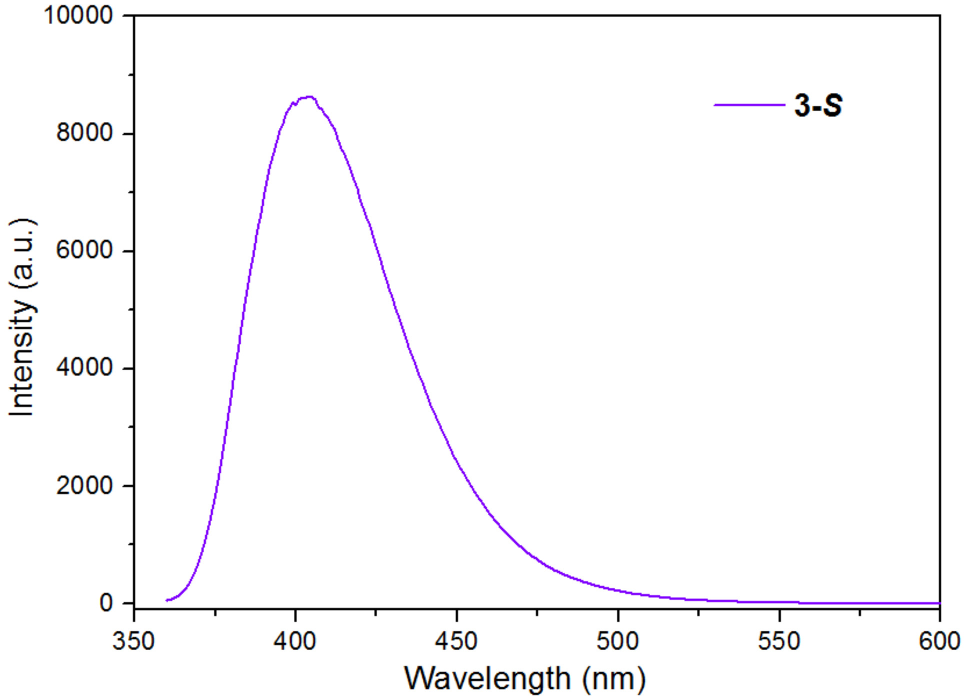

Figure S16. Fluorescence spectrum of **3-*S***. Fluorescence studies were performed using a F-4600 FL Spectrophotometer. **3-*S*** was dissolved in THF in screw-top 1.0 cm quartz cuvettes. The concentration was 1 × 10^{-3} M, the solution was irradiated at 347 nm.

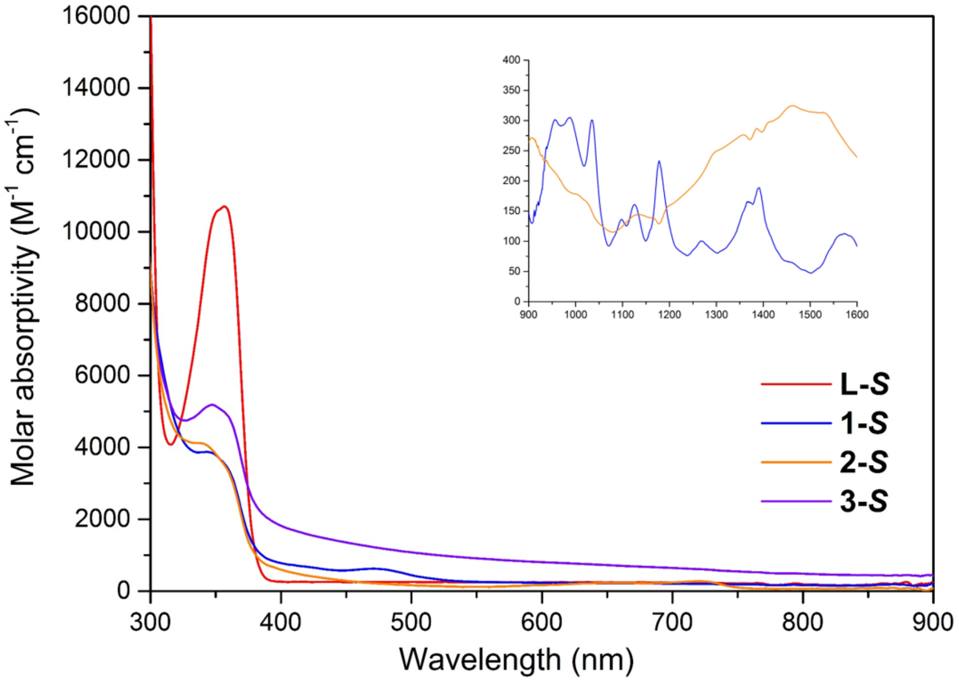

Figure S17. UV-Vis-NIR absorption spectra of complexes **L-*S***, **1-*S*** ~ **3-*S*** measured in THF at RT.

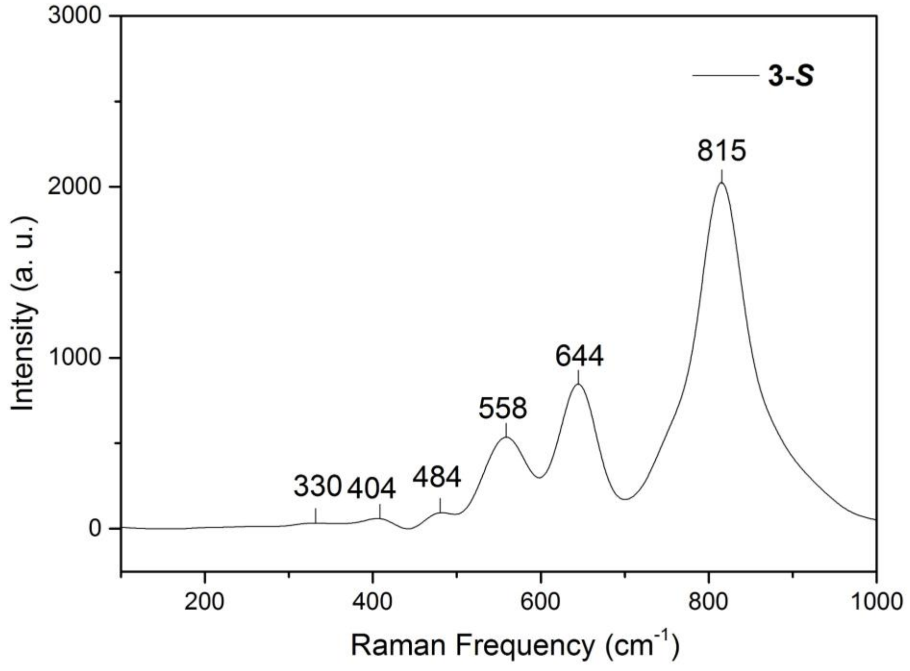

Figure S18. Raman spectrum for complex **3-*S***.

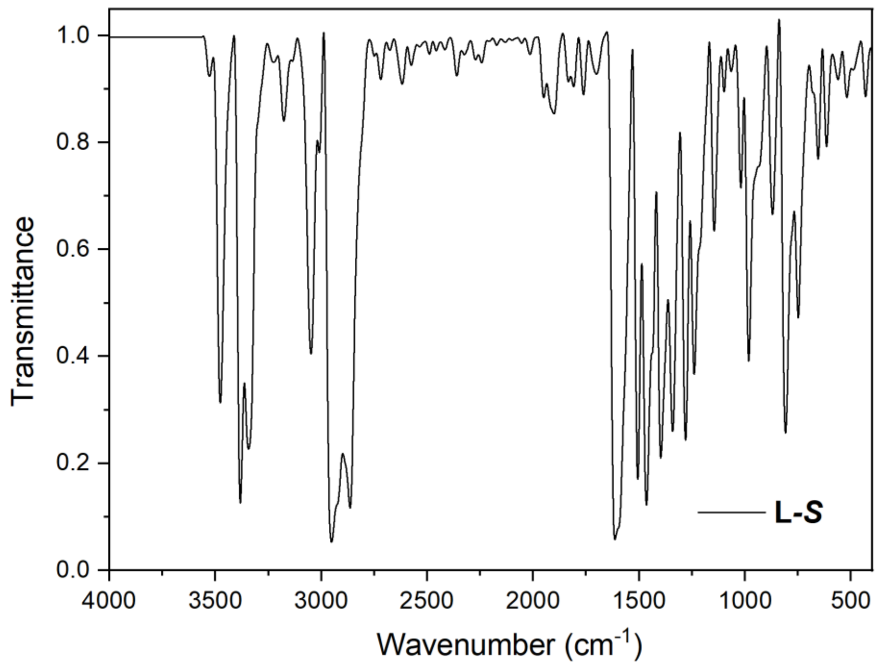

Figure S19. FT-IR spectrum of complex **L-*S*** measured as solid in a KBr pellet at RT.

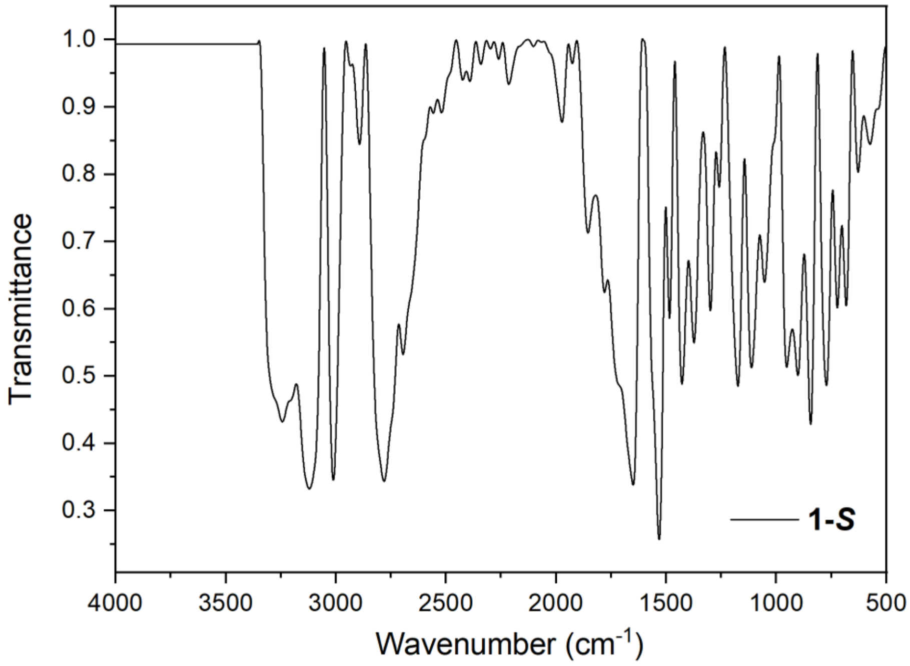

Figure S20. FT-IR spectrum of complex **1-*S*** measured as solid in a KBr pellet at RT.

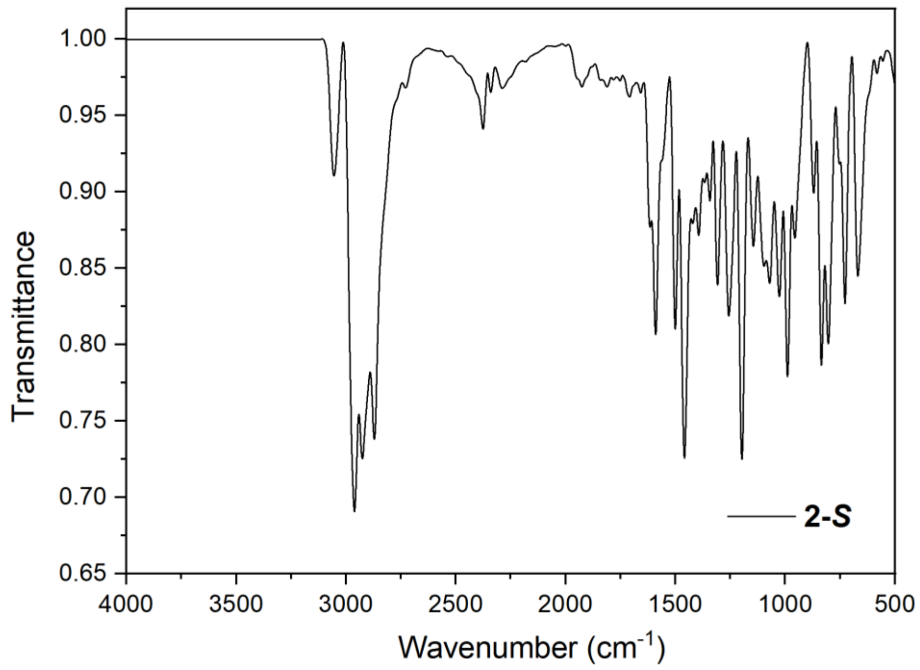

Figure S21. FT-IR spectrum of complex **2-*S*** measured as solid in a KBr pellet at RT.

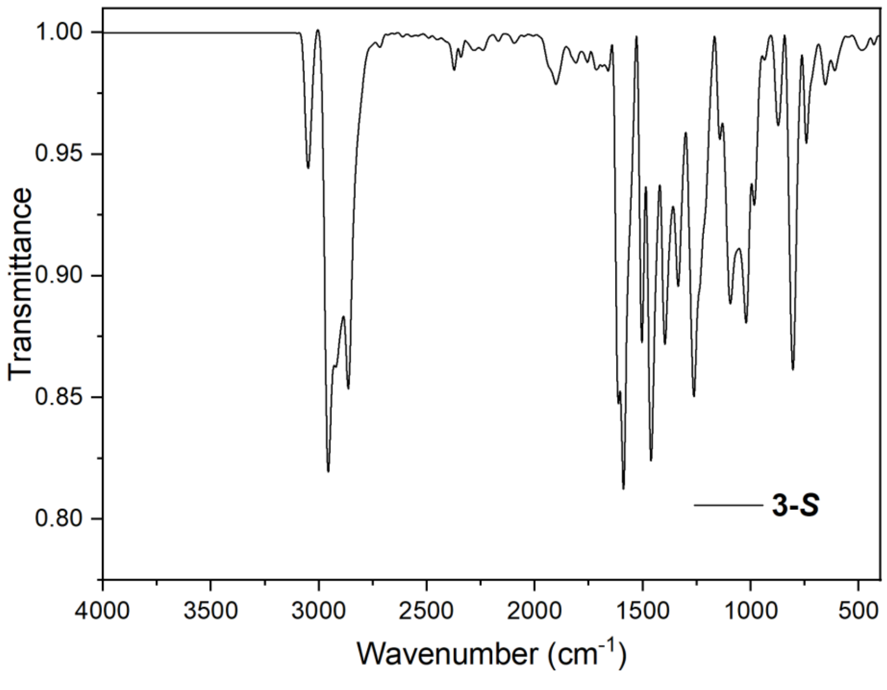

Figure S22. FT-IR spectrum of complex **3-*S*** measured as solid in a KBr pellet at RT.

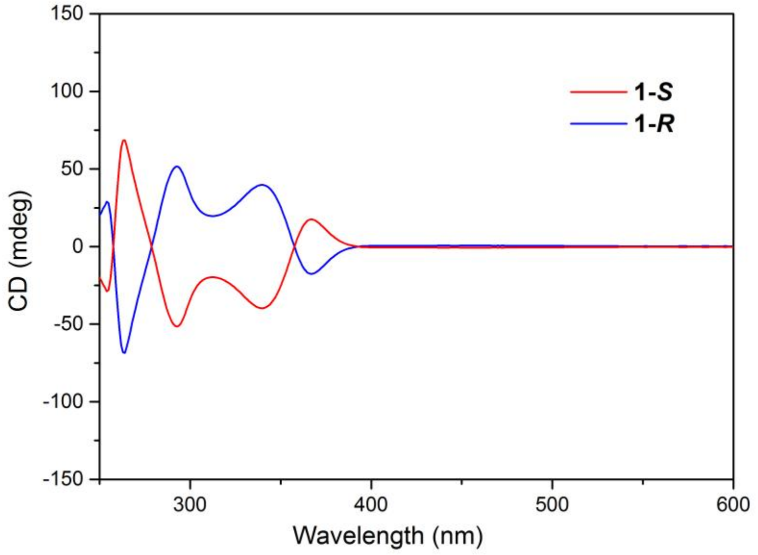

Figure S23. CD spectra of complexes **1-*R*** and **1-*S*** measured in THF at RT.

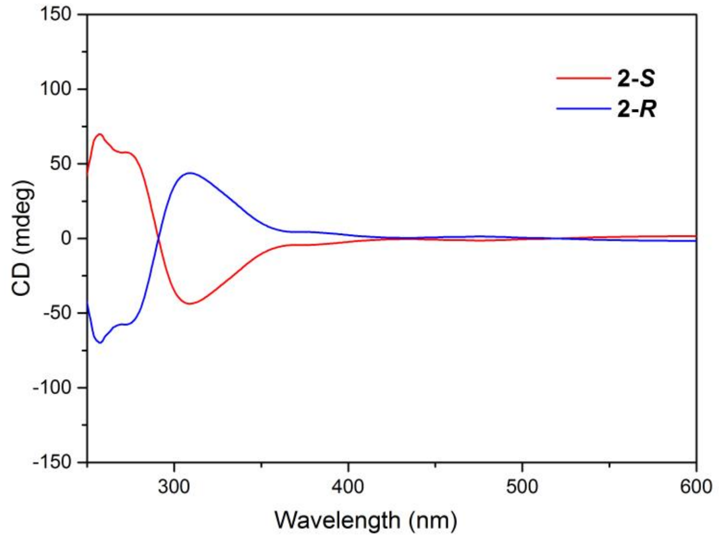

Figure S24. CD spectra of complexes **2-*R*** and **2-*S*** measured in THF at RT.

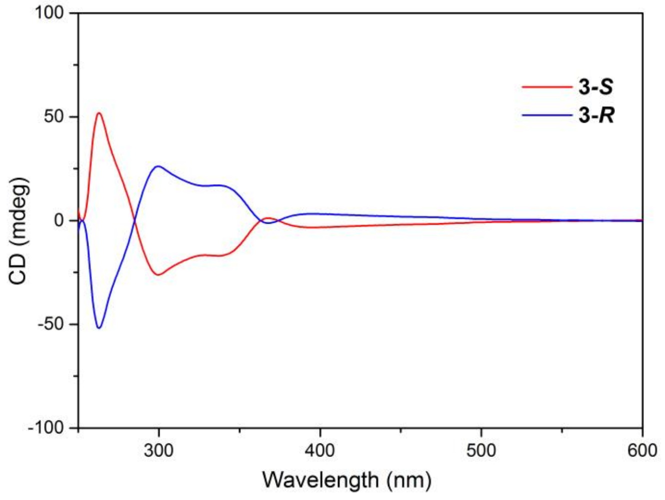

Figure S25. CD spectra of complexes **3-R** and **3-*S*** measured in THF at RT.

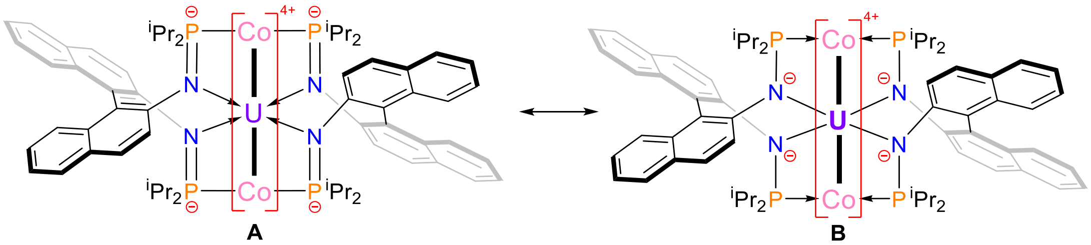

Figure S26. Resonance structures of complex **3-*S***. The overall charge neutrality of complex **3-*S*** arises from the following resonance contributions: Resonance form **A** (the nitrogen atoms act as dative donors to uranium) and resonance form **B** (the nitrogen atoms engage in covalent bonding with uranium).

Table S1. Reported examples of U-Co bonds. The coordination number (CN) plays a crucial role in determining the bonding multiplicity of U-Co bonds. For instance, a 4-coordinated Co center results in a U-Co single bond, whereas a 3-coordinated Co center leads to a U-Co triple bond. In our study, the CN of both Co centers is 2, which gives rise to cumulative quintuple bonds.

	CN. of Co	OS. of Co	U-Co distance	Bonding multiplicity	Ref
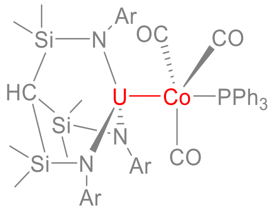	4	0	2.9450(9)	Single bond	*Angew. Chem. Int. Ed.* 2011, *50*, 10388
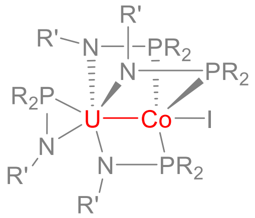	4	+1	2.874(3) ~ 3.0812(7)	Single bond	*Inorg. Chem.* 2013, *52*, 12170
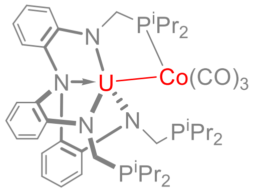	4	-1	3.0319(7)	Single bond	*J. Am. Chem. Soc.* 2014, *136*, 3647
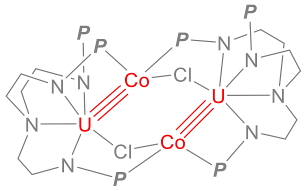	3	0	2.0854(5)	Triple bond	*Chem* 2022, *8*, 1361–1375
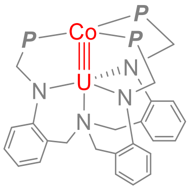	3	-1	2.1890(11)	Triple bond	*Angew. Chem. Int. Ed.* 2025, e202516858
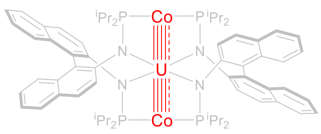	2	-1	2.064(2) ~ 2.1260(16)	Cumulative quintuple bonds	*This work*

3. X-ray crystallographic analysis

Crystals of complexes **1-*S***, **1-*R***, **2-*R***, **3-*S***, and **3-*R*** were successfully grown from a saturated solution stored at room temperature. Despite repeated attempts, suitable crystals of complex **2-*S*** for X-ray diffraction could not be obtained. The crystallographic data for complexes **1-*S***, **1-*R***, **2-*R***, **3-*S***, and **3-*R*** were collected on Bruker D8 venture photon II detectors at 100 K or 193 K with a radiation source of Ga(Kα) (λ = 1.34139 Å) or Mo(Kα) (λ = 0.71073 Å) using the ω-scan technique. Multiscan or empirical absorption corrections (SADABS) were applied. The structures were solved by direct methods, expanded by difference Fourier syntheses, and refined by full-matrix least squares on F^2 using the olex2.[1,2] All non-hydrogen atoms were refined on F^2 by full-matrix least-squares procedures with the use of anisotropic displacement parameters. Hydrogen atoms were introduced at their geometric positions and refined as riding atoms. Evaluation of the crystallographic information file (CIF) using the CheckCIF routine at www.checkcif.iucr.org yielded one B alert for complexes **1-*S*** and **3-*S*** and no A or B alerts for complexes **1-*R***, **2-*R***, and **3-*R***. Response to these alerts are given in Table S2. CCDC 2388408 (**1-*R***), 2388409 (**1-*S***), 2388410 (**2-*R***), 2388411 (**3-*R***), and 2388412 (**3-*S***) contain the crystallographic data for this paper. These data can be obtained free of charge from The Cambridge Crystallographic Data Centre via www.ccdc.cam.ac.uk/data-request/cif. Details of the data collection and refinement for complexes **1-*S***, **1-*R***, **2-*R***, **3-*S***, and **3-*R***, are given in Tables S3 ~ S4.

Table S2. Response to the questions raised in the CIF report of **1-*S*** and **3-*S***.

**Alert level B in 1-*S***	Missing # of FCF Reflection(s) Below Theta(Min). 12 Note	The low angle reflections are probably missing due to the beamstop. We believe the key findings of the structural feature of the complex are unambiguous.

**Alert level B in 3-*S***	Low Bond Precision on C-C Bonds .... 0.027 Ang.	The crystal does not diffract well at high angles, and some disordered ^{i}Pr groups contribute to this B-level alert. However, this is the best data obtained after multiple recrystallization attempts, and the key structural features of the complex are clear and unambiguous.

Table S3. Crystal data and structural refinements for **1-*S***, **1-*R*** and **2-*R***

Complex	**1-*S***	**1-*R***	**2-*R***
empirical formula	$C_{64}H_{80}N_4P_4U$	$C_{64}H_{80}N_4P_4U$	$C_{64}H_{80}Cl_4Co_2N_4P_4U$
formula weight	1267.23	1267.23	1526.89
temperature, K	170.00	100.00	193.15
crystal system	triclinic	monoclinic	orthorhombic
space group	P1	$P2_1$	$P2_12_12_1$
a, Å	16.711(4)	16.5714(6)	14.3986(18)
b, Å	20.509(5)	20.3649(7)	24.848(3)
c, Å	21.697(6)	21.5770(8)	26.602(3)
α, °	89.963(9)	90	90
β, °	79.081(9)	101.1890(10)	90
γ, °	89.942(9)	90	90
V, Å3	7302(3)	7143.3(4)	9518(2)
Z	4	4	4
*d*calcd, g cm^{-3}	1.153	1.178	1.066
μ, mm^{-1}	2.345	2.397	2.250
F(000)	2576.0	2576.0	3064.0
crystal size, mm	0.12 × 0.1 × 0.09	0.16 × 0.13 × 0.12	0.12 × 0.1 × 0.09
2*θ* range/deg	3.944 to 50.7	3.848 to 55.084	4.168 to 50.7
Index ranges	$-20 \le h \le 20$, $-24 \le k \le 24$, $-26 \le l \le 26$	$-21 \le h \le 21$, $-26 \le k \le 26$, $-28 \le l \le 28$	$-17 \le h \le 17$, $-29 \le k \le 29$, $-32 \le l \le 32$

reflns collected	156224	181212	99068
indep reflns	48956 [R_{int} = 0.0830, R_{sigma} = 0.0980]	32682 [R_{int} = 0.0677, R_{sigma} = 0.0574]	17373 [R_{int} = 0.0853, R_{sigma} = 0.0594]
data/restraints/params	48956/9/2694	32682/1/1348	17373/79/759
goodness-of-fit on F^2	1.125	1.055	1.041
final R ($I > 2\sigma(I)$)	R_1 = 0.0485, wR_2 = 0.1232	R_1 = 0.0342, wR_2 = 0.0837	R_1 = 0.0473, wR_2 = 0.1201
R indices (all data)	R_1 = 0.0589, wR_2 = 0.1297	R_1 = 0.0414, wR_2 = 0.0865	R_1 = 0.0552, wR_2 = 0.1259
Largest diff. peak and hole [e·Å^{-3}]	1.40/-0.81	0.87/-0.62	1.02/-0.69
Flack parameter	0.138(5)	0.055(4)	0.087(6)

Table S4. Crystal data and structural refinements for **3-*S*** and **3-*R***

Complex	**3-*S***	**3-*R***
empirical formula	$C_{64}H_{80}Co_2N_4P_4U$	$C_{64}H_{80}Co_2N_4P_4U$
formula weight	1385.09	1385.09
temperature, K	193.15	193.00
crystal system	monoclinic	monoclinic
space group	$P2_1$	$P2_1$
a, Å	11.5953(8)	12.428(2)
b, Å	18.0112(12)	21.316(4)
c, Å	14.8777(10)	23.294(4)
α, °	90	90
β, °	92.036(2)	92.059(6)
γ, °	90	90
V, Å3	3105.2(4)	6166.8(18)

Z	2	4
$d$calcd, g cm^{-3}	1.481	1.492
μ, mm^{-1}	3.274	3.297
F(000)	1396.0	2792.0
crystal size, mm	0.12 × 0.1 × 0.09	0.12 × 0.1 × 0.09
2θ range/deg	4.18 to 55.136	3.772 to 52.818
Index ranges	$-15 \le h \le 15$, $-23 \le k \le 21$, $-19 \le l \le 19$	$-15 \le h \le 15$, $-26 \le k \le 26$, $0 \le l \le 29$
reflns collected	57279	25095
indep reflns	13570 [R_{int} = 0.0941, R_{sigma} = 0.0820]	25095 [R_{sigma} = 0.0384]
data/restraints/params	13570/305/766	25095/529/1594
goodness-of-fit on F^2	0.997	1.040
final R ($I > 2\sigma(I)$)	R_1 = 0.0593, wR_2 = 0.1393	R_1 = 0.0370, wR_2 = 0.0916
R indices (all data)	R_1 = 0.1111, wR_2 = 0.1639	R_1 = 0.0446, wR_2 = 0.0996
Largest diff. peak and hole [e·Å^{-3}]	1.30/-1.29	1.38/-0.94
Flack parameter	0.068(12)	-0.0047(19)

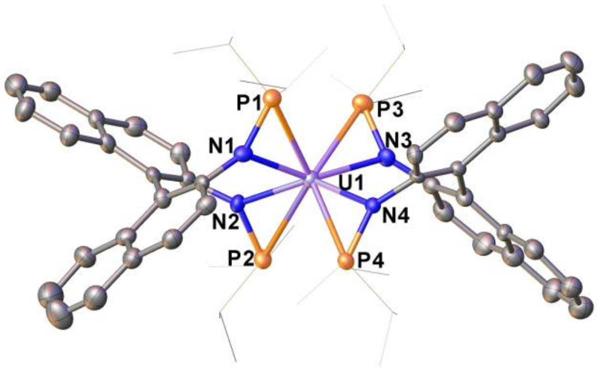

Figure S27. Molecular structure of **1-*S*** in the solid state. Thermal ellipsoids are drawn at 50% probability. P^iPr_2 groups shown as wireframe and H atoms omitted for clarity.

Table S5. Selected bond lengths (Å) for **1-*S***

U1-N1	2.333(9)	U1-N2	2.313(9)
U1-N3	2.357(9)	U1-N4	2.283(8)
N1-P1	1.657(9)	N2-P2	1.657(9)
N3-P3	1.647(10)	N4-P4	1.646(9)

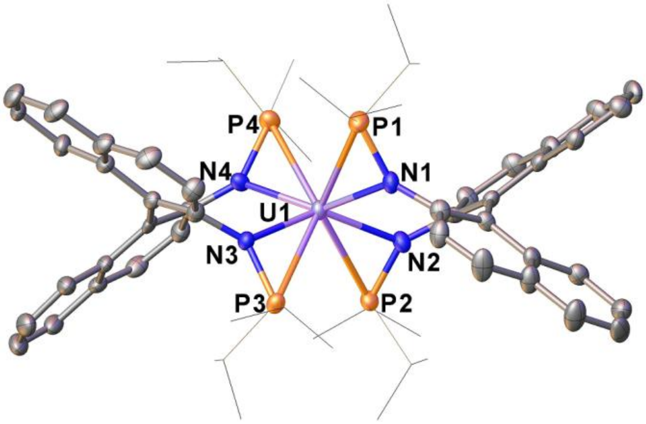

Figure S28. Molecular structure of **1-*R*** in the solid state. Thermal ellipsoids are drawn at 50% probability. P^iPr_2 groups shown as wireframe and H atoms omitted for clarity.

Table S7. Selected bond lengths (Å) for **1-*R***

U1-N1	2.314(5)	U1-N2	2.325(5)
U1-N3	2.274(5)	U1-N4	2.304(5)
N1-P1	1.657(5)	N2-P2	1.664(5)
N3-P3	1.672(6)	N4-P4	1.675(5)

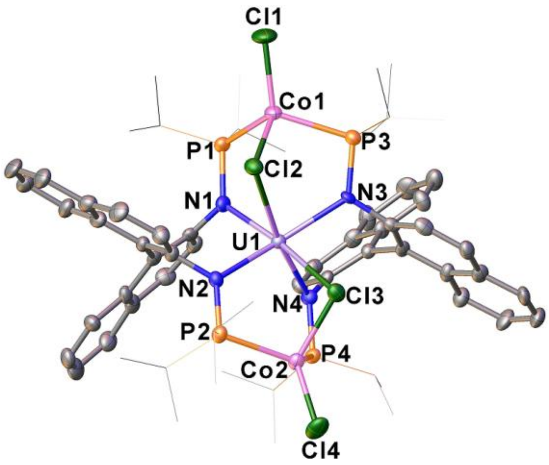

Figure S29. Molecular structure of **2-*R*** in the solid state. Thermal ellipsoids are drawn at 50% probability. P^iPr_2 groups shown as wireframe and H atoms omitted for clarity.

Table S7. Selected bond lengths (Å) for **2-*R***

U1-N1	2.309(7)	U1-N2	2.338(8)
U1-N3	2.322(7)	U1-N4	2.304(7)
N1-P1	1.675(8)	N2-P2	1.693(8)
N3-P3	1.678(7)	N4-P4	1.679(8)
Co1-P1	2.362(3)	Co1-P3	2.348(3)
Co2-P2	2.360(3)	Co2-P4	2.379(3)

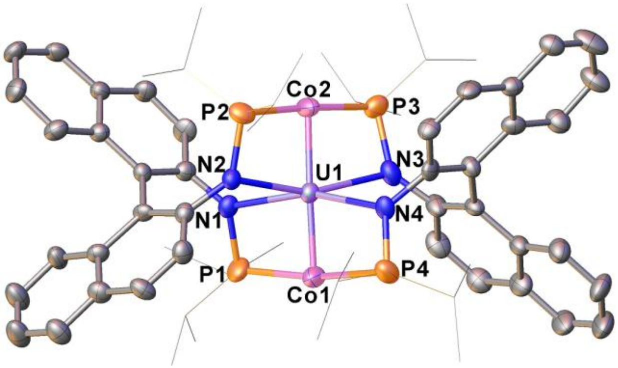

Figure S30. Molecular structure of **3-*S*** in the solid state. Thermal ellipsoids are drawn at 50% probability. P^iPr_2 groups shown as wireframe and H atoms omitted for clarity.

Table S8. Selected bond lengths (Å) and angle(°) for **3-*S***

U1-Co1	2.064(2)	U1-Co2	2.079(2)
U1-N1	2.452(11)	U1-N2	2.441(11)

U1-N3	2.484(14)	U1-N4	2.465(11)
N1-P1	1.640(12)	N2-P2	1.641(12)
N3-P3	1.670(16)	N4-P4	1.612(13)
Co1-P1	2.515(6)	Co1-P4	2.872
Co2-P2	2.329(6)	Co2-P3	2.374(7)
Co1-U1-Co2	175.37(18)	P2-Co2-P3	176.8(2)
P1-Co1-P4	168.908		

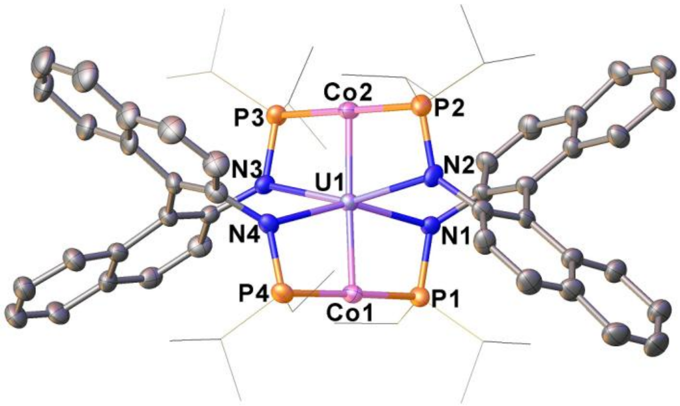

Figure S31. Molecular structure of **3-*R*** in the solid state. Thermal ellipsoids are drawn at 50% probability. P^iPr_2 groups shown as wireframe and H atoms omitted for clarity.

Table S9. Selected bond lengths (Å) and angle(°) for **3-*R***

U1-Co1	2.1260(16)	U1-Co2	2.1215(15)
U1-N1	2.416(8)	U1-N2	2.426(8)
U1-N3	2.418(9)	U1-N4	2.405(7)
N1-P1	1.647(10)	N2-P2	1.657(9)
N3-P3	1.643(9)	N4-P4	1.650(8)
Co1-P1	2.215(3)	Co1-P4	2.220(3)
Co2-P2	2.222(3)	Co2-P3	2.198(3)
Co1-U1-Co2	177.91(7)	P2-Co2-P3	176.42(13)
P1-Co1-P4	179.46(16)		

4. Computational details. All the calculations were performed with the Gaussian 16 program package[3] and OpenMolcas 25.02.[4] Geometry optimizations and frequency calculations, including Raman analysis, were performed at the level of PBE0-D3.[5,6] The small-core Stuttgart relativistic effective core potentials and the corresponding valence basis sets were used for Co (triple-ζ) and U (quadruple-ζ) atoms,[7-11] while the 6-31g* basis sets were utilized for the remaining atoms.[12-16] Based on the optimized structures, we further carried out the single point calculations to obtain better wavefunctions for the following analyses: the canonical molecular orbital (CMO) analysis, the fragment alignment molecular orbital (FAMO) analysis,[17-19] and the principal interacting orbital (PIO) analysis.[20,21] In these single-point calculations, Co and U atoms were also represented by the small-core Stuttgart relativistic effective core potentials and the corresponding valence basis sets,[7-11] whereas the remaining atoms were calculated with 6-311g** basis sets.[15,22,23] Additionally, the PBE0 functional augmented by D3 dispersion corrections was again used in these computations.[5,6] For time-dependent density-functional theory (TDDFT), the calculations were performed with the SMD solvation model.[24]

Moreover, for the complete active space self-consistent field (CASSCF) and restricted active space self-consistent field (RASSCF) calculations, ANO-RCC-VTZP basis sets were used for Co, U, N, and P,[25-27] while ANO-RCC-MB basis sets were used for C and H to make multi-reference calculations computationally tractable.[28] Notably, the input files for OpenMolcas were generated with the help of molecular orbital KIT (MOKIT) software packages.[29,30]

The plots of structures were displayed via GaussView 6.0,[31] and the orbital figures were plotted by IQmol. It should be noted that the isovalues used in IQmol are expressed in a program-specific unit, not the standard atomic unit. Furthermore, as the orbital plots were obtained using various distinct computational and wavefunction analysis methods, we adopted different isovalues to clearly visualize the key orbital features.

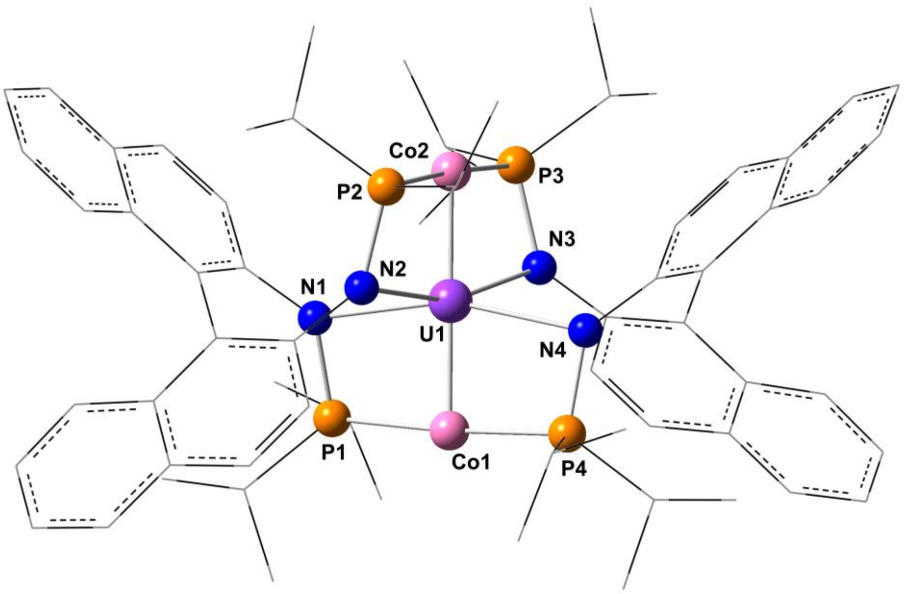

Figure S32. Optimized structure of **3-*S***. For clarity, hydrogen atoms are omitted, isopropyl and binaphthyl moieties are shown in wireframes.

Table S10. Selected bond lengths (Å) for the optimized structure of **3-*S***

U1-Co1	2.06	U1-Co2	2.06
U1-N1	2.36	U1-N2	2.36
U1-N3	2.36	U1-N4	2.36
Co1-P1	2.22	Co1-P4	2.22
Co2-P2	2.22	Co2-P3	2.22
N1-P1	1.67	N2-P2	1.67
N3-P3	1.67	N4-P4	1.67

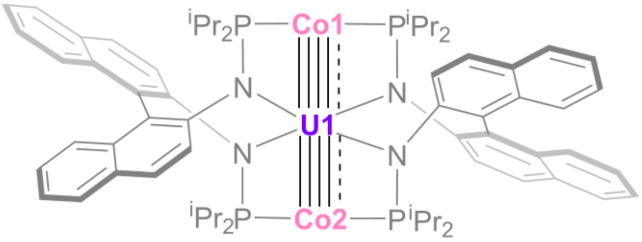

3-*S*

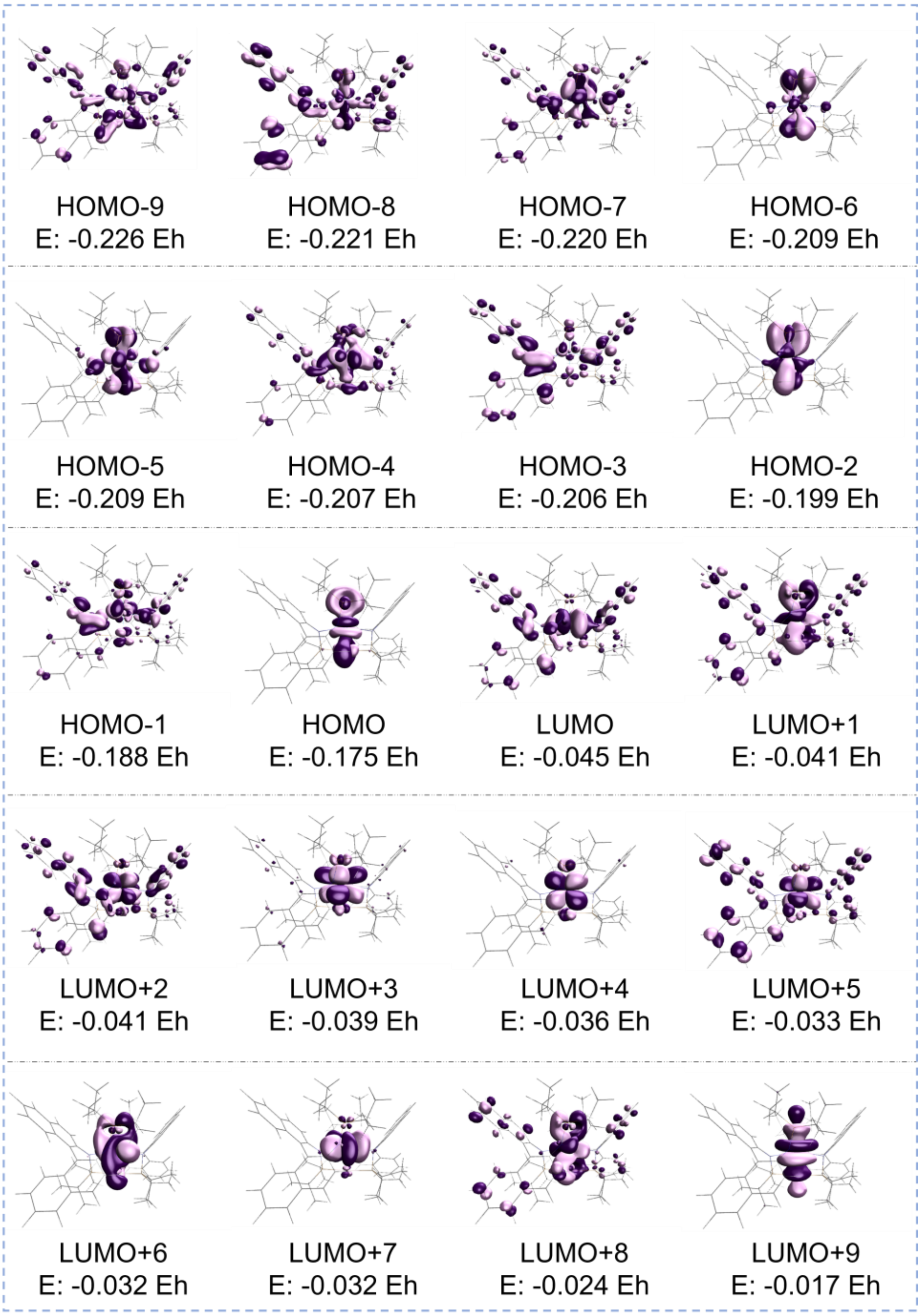

Figure S33. Selected canonical molecular orbitals (CMOs) for **3-*S*** (isovalue = 0.100) with corresponding orbital energies. HOMO stands for the highest occupied molecular orbital, while LUMO denotes the lowest unoccupied molecular orbital. For LUMO+1, LUMO+6, LUMO+8, and LUMO+9, Supplementary Fig. S34 provides clearer plots at a higher isovalue.

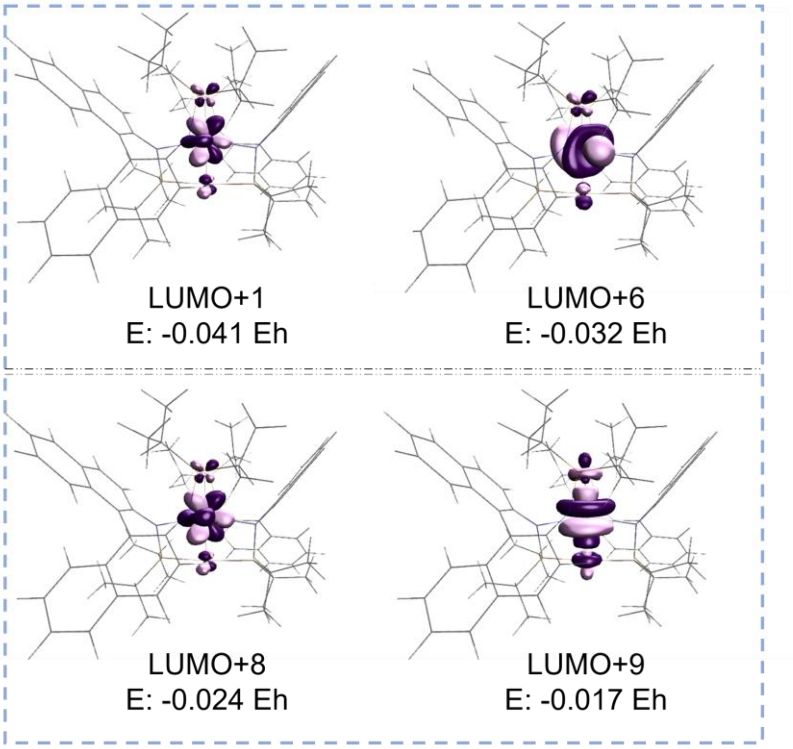

Figure S34. LUMO+1, LUMO+6, LUMO+8, and LUMO+9 for **3-*S*** (isovalue = 0.250) with corresponding orbital energies.

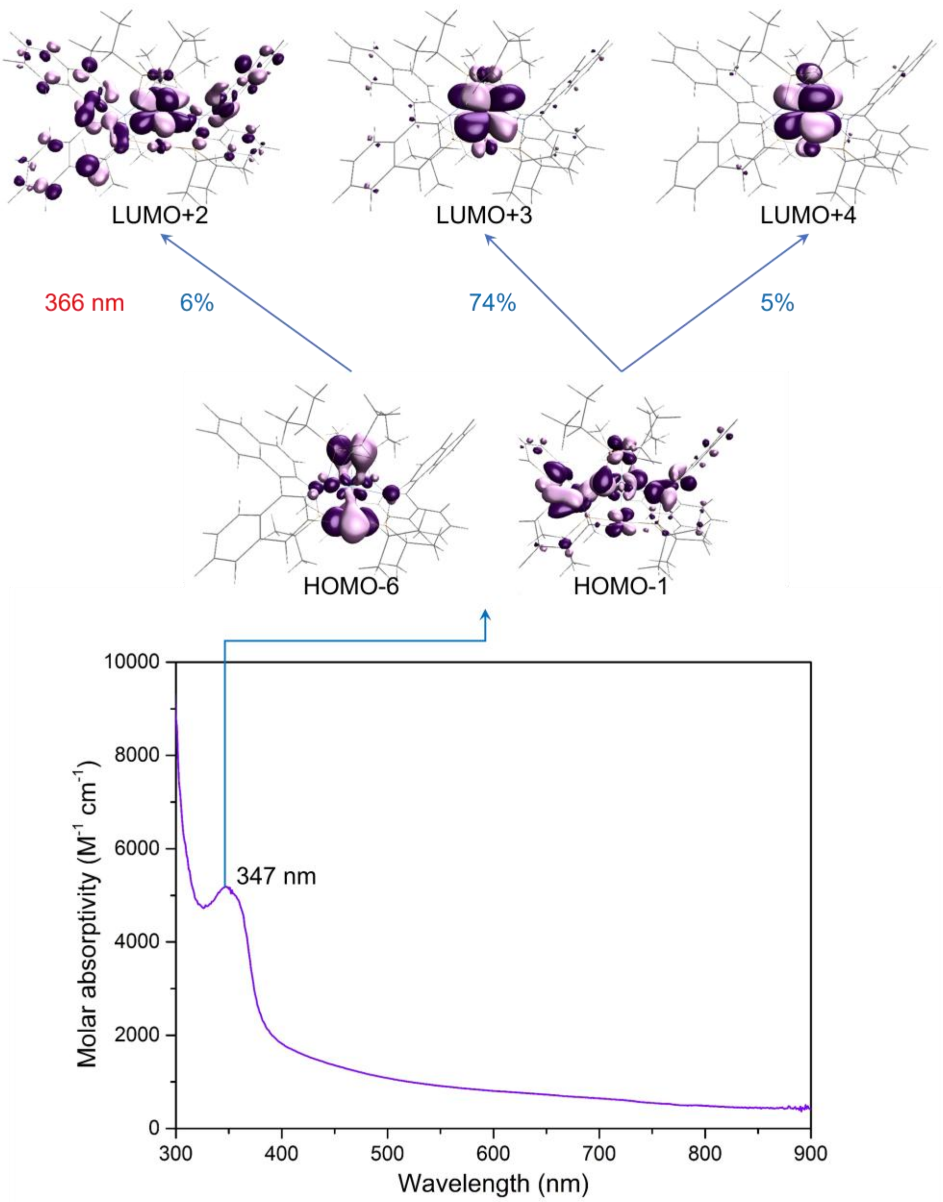

Figure S35. Experimental UV-Vis absorption spectrum of **3-*S*** and calculated electronic transitions (percentage ≥ 5%), along with their percentage contributions to the absorption. The absorption peak of experimental results is at 347 nm, while the absorption peak of TDDFT results is at 366 nm.

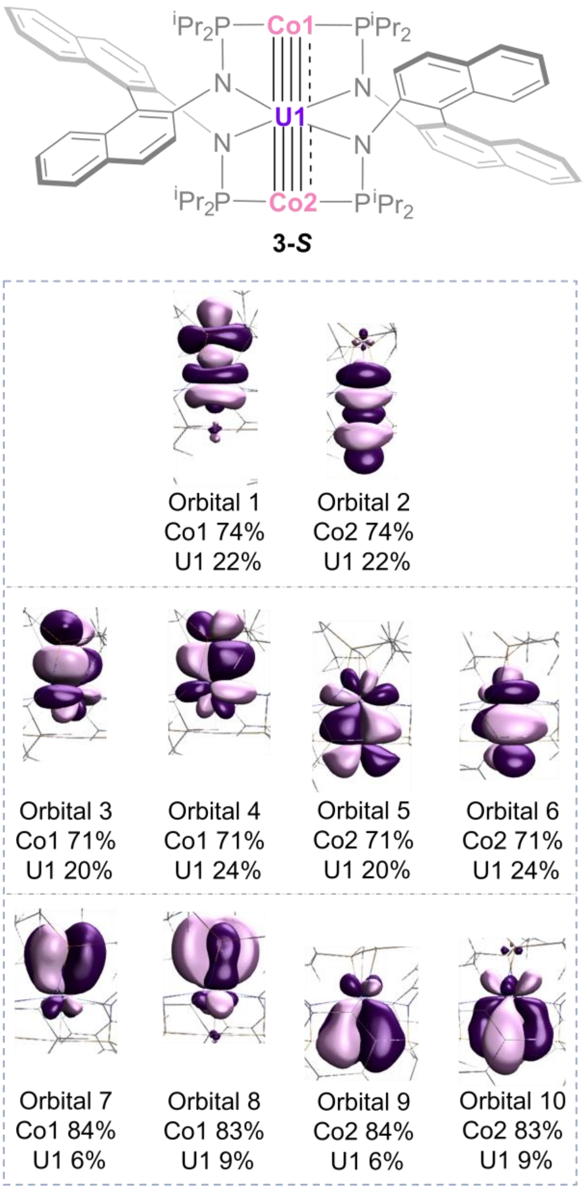

Figure S36. Pure metallic localized orbitals for **3-*S*** (isovalue = 0.100), classified as σ (top row), π (middle row), and δ (bottom row) types, with corresponding Hirshfeld atomic compositions (>5%).

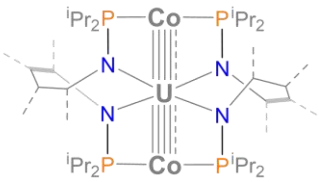

3-*S*

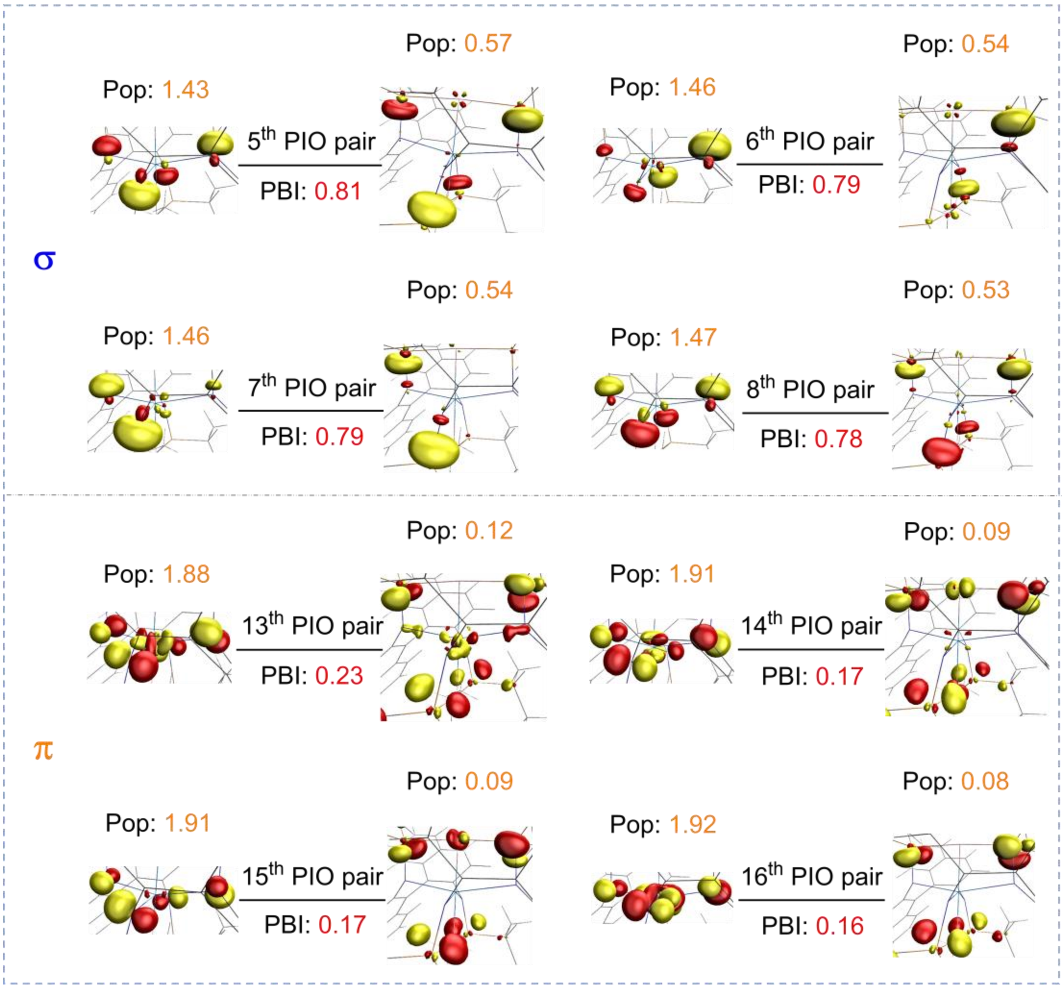

Figure S37. Selected principal interacting orbital pairs of **3-*S*** (isovalue = 0.250) with corresponding populations (orange font) and PIO-based bond indexes (red font). The selected PIO pairs represent the N-P bonding interactions, classified as σ (top two rows) and π (bottom two rows) types.

Table S11. Calculated bond orders of the U–Co bond of **3-*S*** by different indices*.

Compound	Fuzzy bond order	Mayer bond order	PIO bond index (order)
**3-*S***	2.86	2.32	2.70<br>(σ 0.84, π 1.46, δ 0.40)

* We calculated the Fuzzy bond order (FBO)[32] and Mayer bond order (MBO)[33] for the U–Co bonds in complexes **3-*S***. The FBO and MBO values for the U–Co bond among complex **3-*S*** are 2.86 and 2.32, respectively (Table S11). The reason that the bond order indices are much lower than 5.00 is that the U-Co bonds are highly polarized. And the calculated bond order indices would always underestimate the bond order of highly polarized bonds.

A vivid example is that although both non-polarized A (50%) – A (50%) bond and highly polarized A (10%) – B (90%) bond should be considered as a single bond, their calculated bond order would be very different. If the A (50%) – A (50%) bond order is 1.00, the A (10%) – B (90%) bond order would be 1 * (10% * 90%) / (50% * 50%) = 0.36. Though their calculated bond orders are distinct, both should be considered as single bonds. Figure S36 shows that the U-Co bonds in **3-*S*** are highly polarized, rendering the low calculated bond order indices.

Therefore, principal interacting orbital (PIO) analysis represents one of the most reliable approaches to elucidate the intrinsic bonding multiplicity of highly polarized bonds. This method allows us to decompose the total computed bond order into individual contributions from σ-, π-, and δ-type bonding orbitals, with detailed results presented in Figure 6 of the main text. The PIO bond orders clearly demonstrate that δ bonds contribute to the cumulative U–Co bonding interactions.

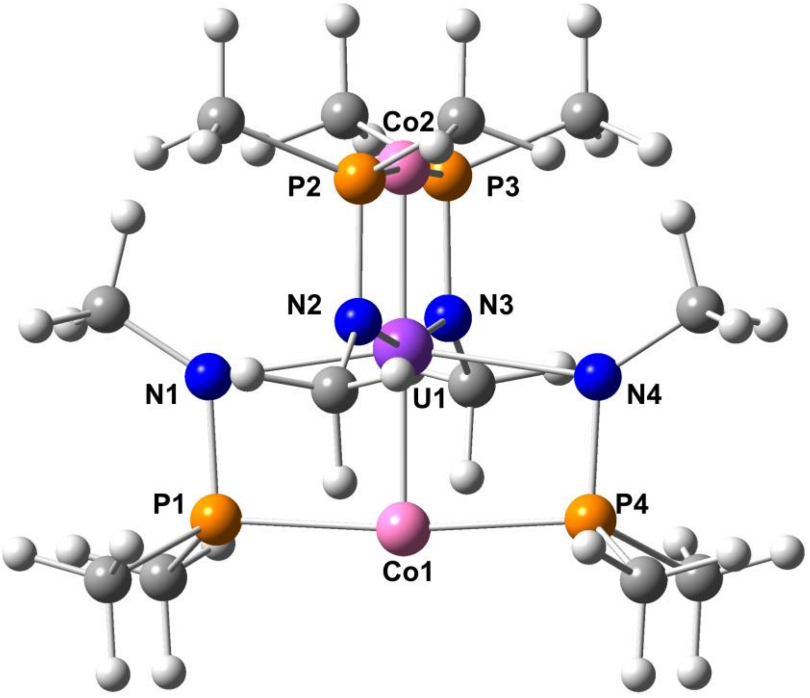

Figure S38. Optimized structure of **3′**. U atom is colored in purple, Co atoms are colored in pink, P atoms are colored in orange, N atoms are colored in blue, C atoms are colored in grey, and H atoms are colored in white.

Table S12. Selected bond lengths (Å) for the optimized structure of **3′**

U1-Co1	2.08	U1-Co2	2.08
U1-N1	2.33	U1-N2	2.33
U1-N3	2.33	U1-N4	2.33
Co1-P1	2.22	Co1-P4	2.22
Co2-P2	2.22	Co2-P3	2.22
N1-P1	1.65	N2-P2	1.65
N3-P3	1.65	N4-P4	1.65

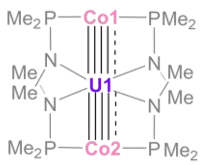

3'

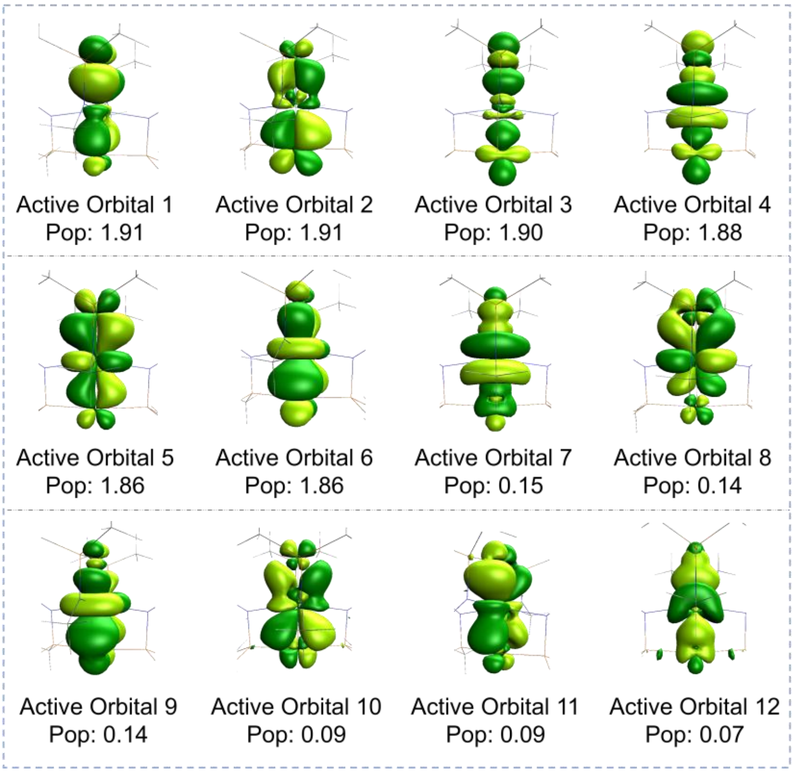

Figure S39. Active orbitals (isovalue = 0.150) with corresponding populations (occupation numbers, rounded to two decimal places), obtained via the complete active space self-consistent field (CASSCF) analysis for **3′**. In the calculations, the active space includes twelve active orbitals and twelve active electrons, denoted as CAS(12,12).

Table S13. The electron configurations (percentage contribution > 1.00%) of **3′**, obtained from the CAS(12,12) calculations

Electron configuration (u: spin up; d: spin down)	Percentage contribution
222222000000	71.76%
22ud22d0000u	1.01%
222220020000	1.45%
222202002000	1.45%

For CASSCF calculations, the active spaces include twelve active orbitals and twelve active electrons, denoted as CAS(12,12). In **3′**, CAS(12,12) contains two σ bonding orbitals, two σ anti-bonding orbitals, four π bonding orbitals, and four π anti-bonding orbitals (Supplementary Fig. S39). The resulting electronic structures possess the dominant electron configuration accounting for 71.8%, with each of the other configurations contributing below 1.5% (Supplementary Table S13).

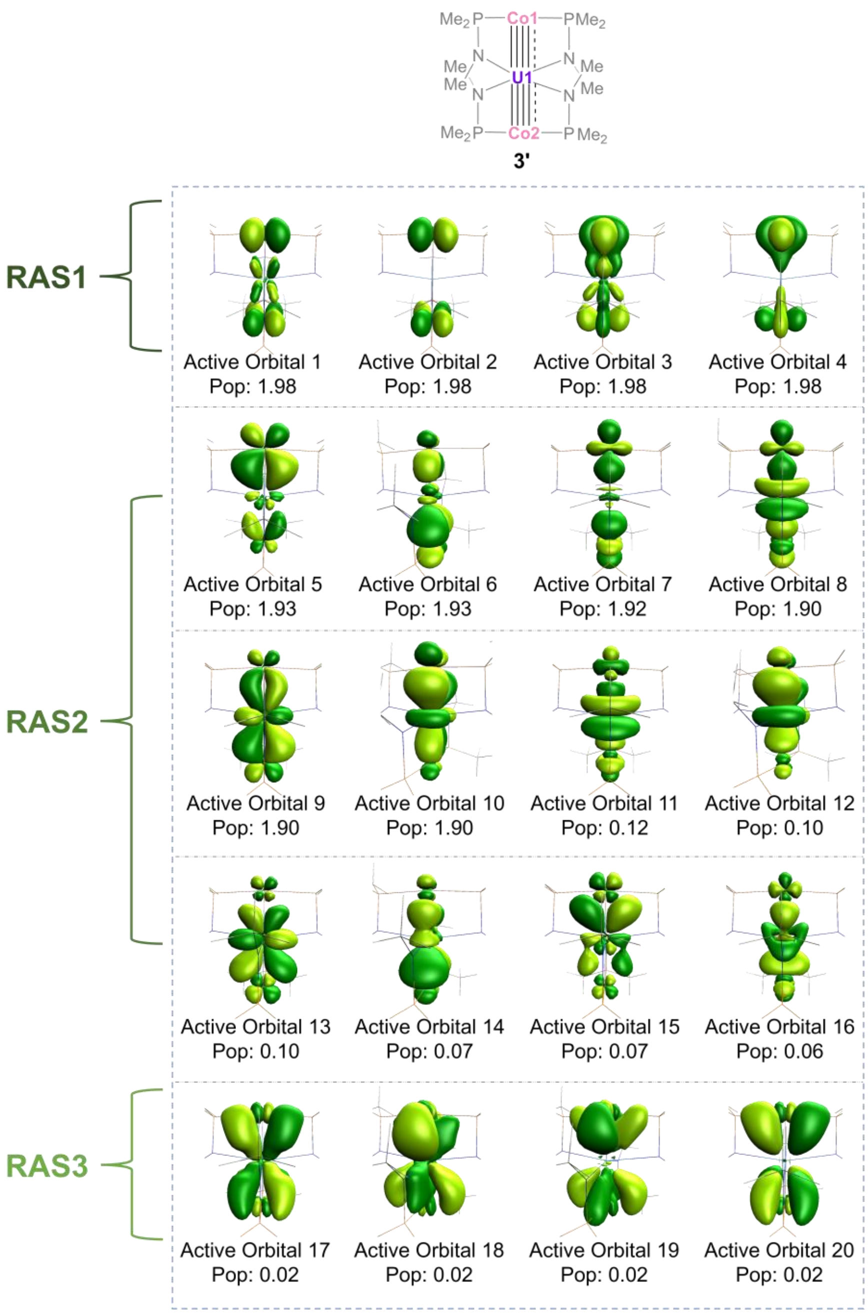

Figure S40. Active orbitals (isovalue = 0.250) with corresponding populations (occupation numbers, rounded to two decimal places), obtained via the restricted active space self-consistent field (RASSCF) analysis for **3′**. The calculations subdivided the active space into three groups, RAS1, RAS2, and RAS3. In RAS1, four orbitals are doubly occupied except for a maximum of one hole allowed in this subspace. In RAS2, twelve orbitals and twelve electrons are included, with all possible occupations permitted. In RAS3, four orbitals are unoccupied except for a maximum of one electron allowed in this subspace. The active space could be denoted as RAS(20,1,1; 4,12,4).

Table S14. The electron configurations (percentage contribution > 1.00%) of **3′**, obtained from the RAS(20,1,1; 4,12,4) calculations

Electron configuration (u: spin up; d: spin down)	Percentage contribution
22222222220000000000	69.48%
222u2222220000000d0	1.22%
22u22222220000000d00	1.26%
u2222222220000000d000	1.37%
2u222222220000000000d	1.10%

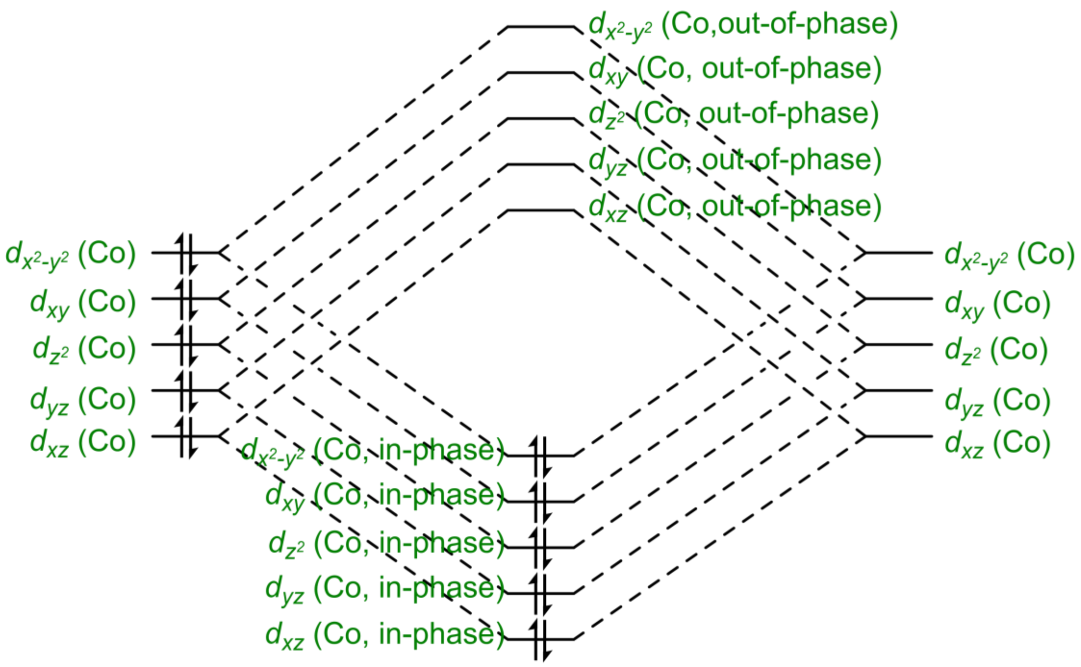

Figure S41. Orbital interaction diagram for the linear combination of *d* (Co) orbitals in RAS(20,1,1; 4,12,4) calculations.

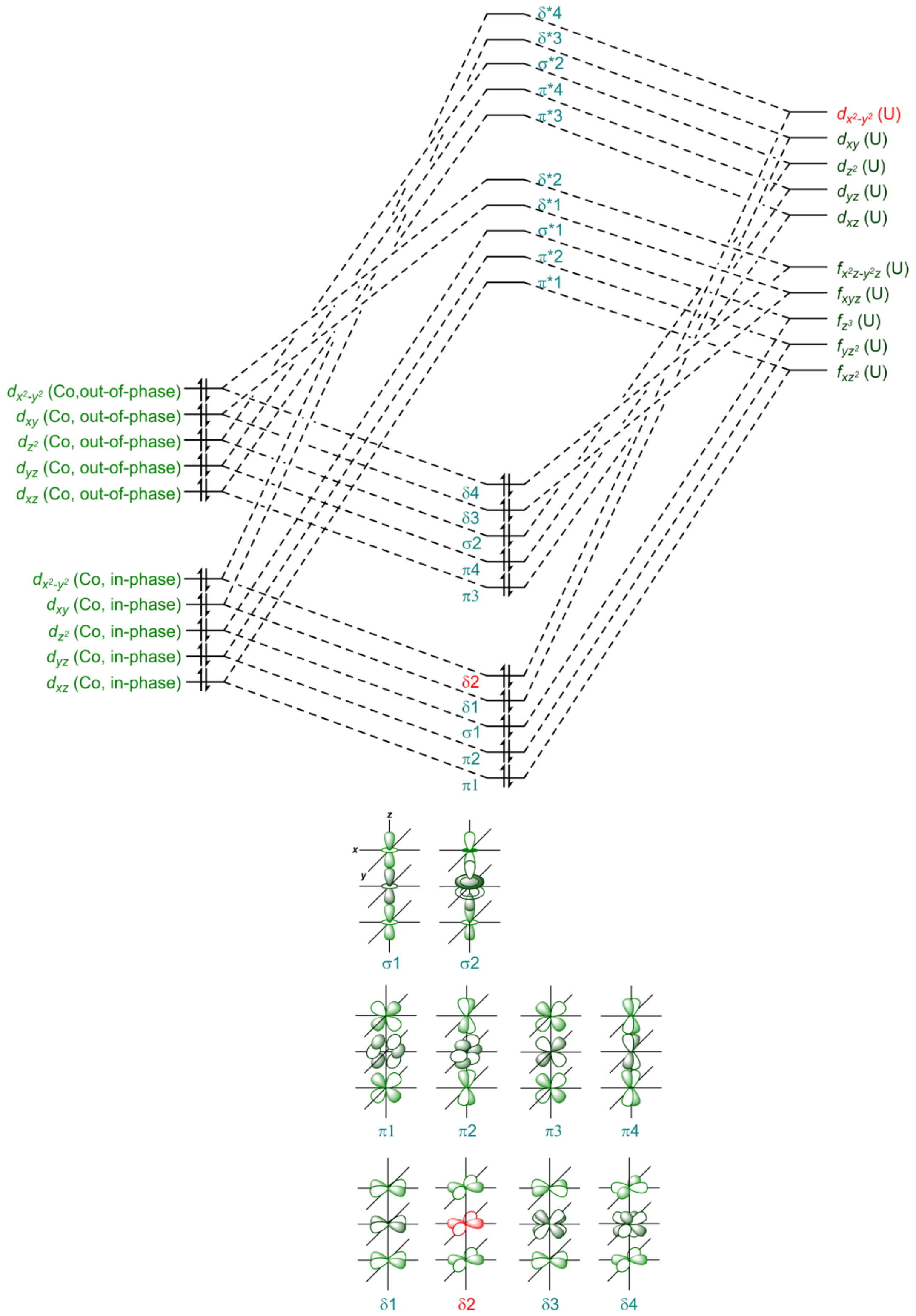

Figure S42. Molecular orbital interaction diagram for RAS(20,1,1; 4,12,4) calculations. The $d_{x^2-y^2}$ (U) orbital would have bonding interactions with the ligated N atoms, leading to the non-bonding δ orbital (active orbital 2) in RAS(20,1,1; 4,12,4) calculations.

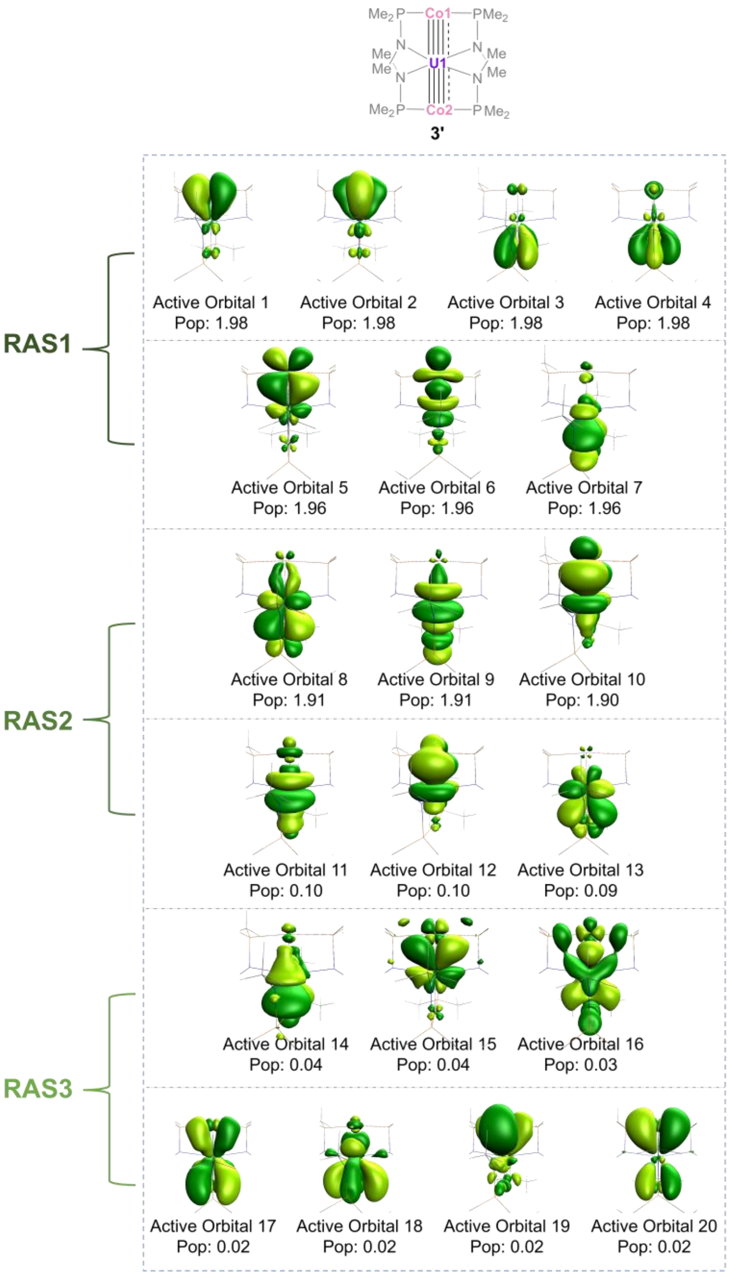

Figure S43. Active orbitals (isovalue = 0.150) with corresponding populations (occupation numbers, rounded to two decimal places), obtained via the restricted active space self-consistent field (RASSCF) analysis for **3′**. The calculations subdivided the active space into three groups, RAS1, RAS2, and RAS3. In RAS1, four orbitals are doubly occupied except for a maximum of two holes allowed in this subspace. In RAS2, twelve orbitals and twelve electrons are included, with all possible occupations permitted. In RAS3, four orbitals are unoccupied except for a maximum of two electrons allowed in this subspace. The active space could be denoted as RAS(20,2,2; 7,6,7).

Table S15. The electron configurations (percentage contribution > 1.00%) of **3′**, obtained from the RAS(20,2,2; 7,6,7) calculations

Electron configuration (u: spin up; d: spin down)	Percentage contribution
22222222220000000000	77.79%
22222222200200000000	2.37%
22222220220020000000	1.46%
22222222022000000000	1.46%
2222222uu2d0d0000000	1.08%

Applying the Pipek-Mezey localization method to the bonding active orbitals obtained from the RAS(20,1,1; 4,12,4) calculations in **3′** revealed localized orbitals with high similarity to the pure metallic localized orbitals (Supplementary Figs. S36 and S44).

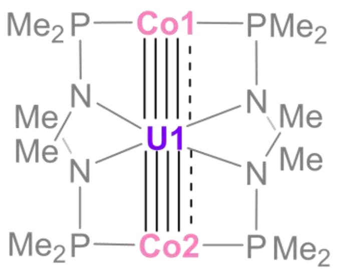

3'

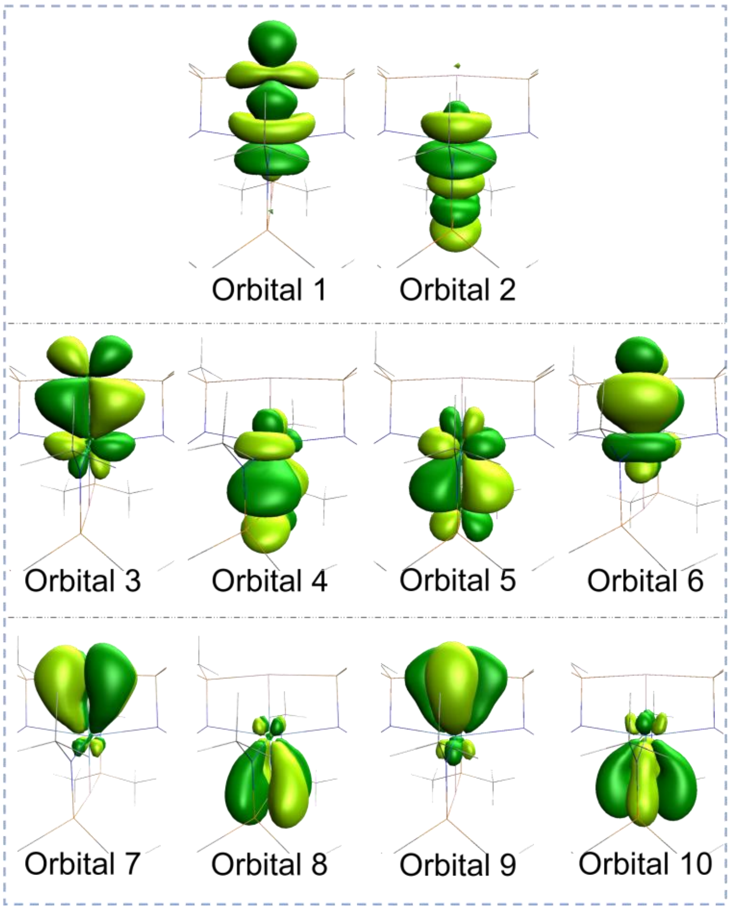

Figure S44. Localized bonding orbitals (isovalue = 0.150), obtained by applying the Pipek-Mezey localization method to the bonding orbitals of **3′** among the RAS(20,1,1; 4,12,4) active space. The orbitals are classified as σ (top row), π (middle row), and δ (bottom row) types.

Optimized coordinates

3-*S*

155

C	-2.95917521	0.43982203	-1.54234611
C	-2.99609221	1.38145510	-2.61267319
H	-2.17815616	1.35384609	-3.32560124
C	-4.00225029	2.29679816	-2.74630620
H	-4.00051329	3.00039522	-3.57613726
C	-5.05075636	2.35158817	-1.79799013
C	-6.08544041	3.31285324	-1.89948714
H	-6.07503245	3.99499629	-2.74702820
C	-7.07434251	3.39321824	-0.95039507
H	-7.86276158	4.13574730	-1.03741408
C	-7.05406651	2.50732818	0.14779301
H	-7.82637054	2.57494918	0.90965807
C	-6.06613544	1.55923811	0.26854702
H	-6.06442842	0.89545307	1.12596708
C	-5.03467536	1.43292310	-0.70267705
C	-3.98050529	0.46481103	-0.58029504
C	-3.97884429	-0.47055903	0.58220004
C	-2.95629221	-0.44436003	1.54285911
C	-2.98686421	-1.39066110	2.60929419
H	-2.16668516	-1.36374110	3.31960524
C	-3.98959329	-2.30976917	2.74226820
H	-3.98296328	-3.01674122	3.56919925
C	-5.04117436	-2.36359417	1.79735413
C	-6.07365542	-3.32712124	1.89967813
H	-6.05874443	-4.01189929	2.74502420
C	-7.06617249	-3.40612725	0.95426607
H	-7.85302154	-4.15023630	1.04198008
C	-7.05169452	-2.51652118	-0.14095401
H	-7.82702957	-2.58267419	-0.89986907
C	-6.06569046	-1.56651211	-0.26261402
H	-6.06885044	-0.90003806	-1.11787508
C	-5.03055136	-1.44146210	0.70484505
C	-2.97300622	-3.08134322	-1.45407910
H	-3.40141425	-2.68439219	-0.52801204
C	-2.55978118	-4.52710233	-1.17587809
H	-3.44091325	-5.10837237	-0.87781306
H	-1.82616213	-4.58290233	-0.36284403
H	-2.12575415	-5.01063336	-2.05794215
C	-4.02909629	-2.98785421	-2.55145918
H	-3.70469626	-3.50507725	-3.46074625

H	-4.26436331	-1.95017314	-2.80971720
H	-4.95633336	-3.46104525	-2.20825216
C	-0.80361106	2.13794915	3.37442924
H	0.02190000	1.41737810	3.27905824
C	-0.19841401	3.51083225	3.65128726
H	-0.97433707	4.27139131	3.79456327
H	0.45422703	3.83491728	2.83158821
H	0.40273103	3.48326025	4.56905433
C	-1.70313912	1.65272312	4.50777732
H	-2.20865716	0.71724205	4.24901631
H	-2.46841418	2.38905717	4.76841134
H	-1.10164808	1.47194211	5.40814841
C	-2.97526021	3.08351022	1.43965410
H	-3.41059024	2.67040819	0.52421704
C	-4.02523529	3.01637722	2.54457818
H	-3.69067826	3.54475126	3.44392025
H	-4.26780931	1.98483214	2.82014820
H	-4.95051536	3.49234025	2.19977816
C	-2.55753918	4.52160432	1.12958708
H	-3.43895625	5.10199937	0.83056906
H	-1.83339713	4.55703033	0.30696802
H	-2.10984215	5.01958136	1.99666214
C	0.79128106	2.13205615	-3.37061524
H	-0.02792000	1.40492510	-3.27021924
C	0.17290201	3.49963125	-3.64458126
H	0.94121307	4.26698931	-3.79231427
H	-0.47812903	3.81805127	-2.82139020
H	-0.43303603	3.46647225	-4.55903133
C	1.68816312	1.65363012	-4.50889133
H	1.08289108	1.46636111	-5.40539939
H	2.20390316	0.72311105	-4.25227131
H	2.44497017	2.39677617	-4.77499535
C	2.97430621	3.08166422	-1.44893310
H	3.40814324	2.67641819	-0.52918604
C	2.56005718	4.52379832	-1.15359308
H	3.44224425	5.10412537	-0.85676906
H	1.83295113	4.56953933	-0.33411402
H	2.11752015	5.01505036	-2.02711914
C	4.02482329	3.00097522	-2.55255518
H	3.69336927	3.52370225	-3.45623225
H	4.26296131	1.96621614	-2.81983620
H	4.95193936	3.47550025	-2.21075216
C	2.95772221	-0.44188803	-1.54152911
C	2.99192022	-1.38659310	-2.60926219

H	2.17256816	-1.36071010	-3.32055124
C	3.99703229	-2.30310217	-2.74231820
H	3.99302629	-3.00898421	-3.57020126
C	5.04751236	-2.35591717	-1.79611313
C	6.08192043	-3.31742824	-1.89790614
H	6.06956446	-4.00135029	-2.74398620
C	7.07307752	-3.39567925	-0.95099407
H	7.86137556	-4.13830630	-1.03827207
C	7.05526451	-2.50738218	0.14525401
H	7.82942056	-2.57314619	0.90540206
C	6.06741944	-1.55922411	0.26637002
H	6.06784643	-0.89371206	1.12241508
C	5.03368036	-1.43494810	-0.70268005
C	3.97991429	-0.46628203	-0.58036904
C	3.97932829	0.46918003	0.58204604
C	5.03139836	1.43961011	0.70449405
C	6.06415145	1.56656211	-0.26527602
H	6.06517943	0.90158707	-1.12174908
C	7.05012853	2.51666918	-0.14430801
H	7.82353759	2.58457819	-0.90502906
C	7.06704849	3.40415625	0.95262607
H	7.85391755	4.14830630	1.03981507
C	6.07686943	3.32317224	1.90033114
H	6.06388544	4.00641729	2.74694820
C	5.04421836	2.35975217	1.79862213
C	3.99454729	2.30420816	2.74560420
H	3.99007729	3.00920922	3.57422926
C	2.99081622	1.38619410	2.61228019
H	2.17193116	1.35788710	3.32409924
C	2.95762921	0.44254403	1.54358411
C	2.97404521	-3.08314122	1.44477610
H	3.40461024	-2.67750619	0.52360704
C	2.55734619	-4.52471332	1.15012608
H	3.43787125	-5.10579137	0.84985406
H	1.82718913	-4.56931833	0.33328502
H	2.11748615	-5.01596636	2.02501615
C	4.02886129	-3.00427621	2.54439918
H	4.95443736	-3.47881925	2.19852816
H	3.70057426	-3.52788325	3.44872325
H	4.26858531	-1.96997414	2.81197620
C	0.79845606	-2.13298015	3.37505425
H	-0.01841900	-1.40265910	3.27888523
C	0.17629301	-3.49885725	3.64903126
H	0.94244907	-4.26924731	3.79209127

H	-0.47925103	-3.81331228	2.82791420
H	-0.42565903	-3.46529925	4.56610833
C	1.70112112	-1.65964312	4.51093433
H	1.09965108	-1.47147511	5.40980341
H	2.21963116	-0.73070405	4.25410730
H	2.45588417	-2.40617417	4.77331935
Co	-0.00076100	-2.06081315	0.00217700
Co	0.00082800	2.06093215	0.00219500
N	-1.83248413	-0.36986703	-1.44169810
N	-1.83283313	0.37001703	1.44374910
N	1.83208313	0.36947903	-1.44210010
N	1.83316313	-0.37025103	1.44347210
P	-1.48906711	-1.98829714	-1.64855812
P	-1.49345811	1.98960114	1.64738412
P	1.49072211	1.98864514	-1.64695012
P	1.49188411	-1.98917514	1.64899312
U	-0.00000500	0.00009100	0.00196500
C	-0.78587706	-2.12700015	-3.37125524
H	0.02355400	-1.38898610	-3.27039724
C	-1.68643412	-1.66233912	-4.51230933
H	-1.08155908	-1.46706111	-5.40737941
H	-2.21660416	-0.73921405	-4.25811731
H	-2.43137718	-2.41690517	-4.77986034
C	-0.14858901	-3.48664625	-3.64144426
H	-0.90585007	-4.26497131	-3.78858127
H	0.50558904	-3.79393627	-2.81653920
H	0.45820003	-3.44711125	-4.55511933

3′

59

C	0.00000000	3.51558325	0.44749803
C	-3.51558325	0.00000000	-0.44749803
C	-1.42091110	3.17164623	-2.65717019
H	-2.34841017	2.73607320	-2.27308416
C	-3.17164623	-1.42091110	2.65717019
H	-2.73607320	-2.34841017	2.27308416
C	-3.17164623	1.42091110	2.65717019
H	-2.73607320	2.34841017	2.27308416
C	3.17164623	1.42091110	2.65717019
H	2.73607320	2.34841017	2.27308416
C	3.17164623	-1.42091110	2.65717019
H	2.73607320	-2.34841017	2.27308416
C	3.51558325	0.00000000	-0.44749803

C	0.00000000	-3.51558325	0.44749803
C	1.42091110	-3.17164623	-2.65717019
H	2.34841017	-2.73607320	-2.27308416
C	-1.42091110	-3.17164623	-2.65717019
H	-2.34841017	-2.73607320	-2.27308416
Co	0.00000000	0.00000000	-2.08350915
Co	0.00000000	0.00000000	2.08350915
N	0.00000000	2.30342917	-0.32759702
N	-2.30342917	0.00000000	0.32759702
N	2.30342917	0.00000000	0.32759702
N	0.00000000	-2.30342917	-0.32759702
P	0.00000000	2.21553716	-1.97707914
P	-2.21553716	0.00000000	1.97707914
P	2.21553716	0.00000000	1.97707914
P	0.00000000	-2.21553716	-1.97707914
U	0.00000000	0.00000000	0.00000000
C	1.42091110	3.17164623	-2.65717019
H	2.34841017	2.73607320	-2.27308416
H	-1.43139711	-3.10984922	-3.75087127
H	-1.37140710	-4.22656931	-2.36240117
H	1.43139711	-3.10984922	-3.75087127
H	1.37140710	-4.22656931	-2.36240117
H	-1.37140710	4.22656931	-2.36240117
H	-1.43139711	3.10984922	-3.75087127
H	1.37140710	4.22656931	-2.36240117
H	1.43139711	3.10984922	-3.75087127
H	-3.10984922	1.43139711	3.75087127
H	-4.22656931	1.37140710	2.36240117
H	-4.22656931	-1.37140710	2.36240117
H	-3.10984922	-1.43139711	3.75087127
H	3.10984922	1.43139711	3.75087127
H	4.22656931	1.37140710	2.36240117
H	3.10984922	-1.43139711	3.75087127
H	4.22656931	-1.37140710	2.36240117
H	0.88910207	4.14207930	0.26479502
H	0.00000000	3.25542123	1.51454011
H	-4.14207930	-0.88910207	-0.26479502
H	-3.25542123	0.00000000	-1.51454011
H	4.14207930	0.88910207	-0.26479502
H	3.25542123	0.00000000	-1.51454011
H	-0.88910207	-4.14207930	0.26479502
H	0.00000000	-3.25542123	1.51454011
H	-0.88910207	4.14207930	0.26479502
H	-4.14207930	0.88910207	-0.26479502

H	4.14207930	-0.88910207	-0.26479502
H	0.88910207	-4.14207930	0.26479502

UCo^{2+}

108

P	-1.59932900	1.88571300	1.42760900
P	-1.60256000	0.29431000	-2.34472800
P	-1.60225600	-2.17806900	0.91909700
N	2.81948700	-0.00065800	-0.00084100
N	1.02504300	1.97268100	0.76573500
N	1.02323600	-0.32033900	-2.09280600
N	1.02238400	-1.65099000	1.32398700
C	3.09326400	1.40054500	-0.32861200
C	4.19486200	1.73294400	-1.10978700
H	4.84520800	0.94339300	-1.47473900
C	4.46738000	3.06590900	-1.38753900
H	5.34266200	3.32898900	-1.97207200
C	3.62642600	4.06199500	-0.88547800
H	3.85031500	5.10620000	-1.08223100
C	2.50163100	3.73885100	-0.14066400
H	1.85637000	4.52713500	0.23349100
C	2.18914800	2.39892800	0.14035200
C	0.00386900	2.87003800	1.26257900
H	-0.18435400	3.69670800	0.56182700
H	0.27604900	3.31219600	2.23182500
C	-1.65743000	1.68313800	3.27700800
H	-1.48931300	2.69013500	3.68274000
C	-0.50024300	0.78441300	3.70954200
H	-0.46624100	0.71358500	4.80214900
H	0.47697700	1.15115500	3.37367500
H	-0.63287200	-0.23020300	3.31619600
C	-2.99806700	1.16747000	3.79164100
H	-2.92632100	0.96475000	4.86599400
H	-3.29414400	0.23769000	3.29686500
H	-3.80385600	1.89267900	3.65280100
C	-2.89593900	3.12454400	0.94968900
H	-2.52747000	3.48713600	-0.02036100
C	-2.99143800	4.31473600	1.90246800
H	-3.68294100	5.05821600	1.49134800
H	-2.02626800	4.81240200	2.04839900
H	-3.37632800	4.02236400	2.88444400
C	-4.24559400	2.44414000	0.71552600
H	-4.94992100	3.15784800	0.27416100

H	-4.69009600	2.08198900	1.64707300
H	-4.15802800	1.59178300	0.03015900
C	3.09056600	-0.98580300	-1.05051200
C	4.19050800	-1.83081100	-0.94812300
H	4.84140100	-1.75317500	-0.08219900
C	4.46079200	-2.73864100	-1.96344600
H	5.33478800	-3.37817700	-1.89917700
C	3.61933200	-2.80037800	-3.07684700
H	3.84158200	-3.49341600	-3.88276900
C	2.49622900	-1.99148200	-3.16919200
H	1.85069300	-2.06023900	-4.03883800
C	2.18586500	-1.07724700	-2.14938400
C	0.00020400	-0.34006100	-3.11645500
H	-0.18815000	-1.36067400	-3.48076600
H	0.27049200	0.27769600	-3.98499300
C	-1.66226200	1.99719200	-3.09404100
H	-1.49535400	1.84466100	-4.16912100
C	-3.00313000	2.69996100	-2.90371800
H	-2.93261900	3.73135000	-3.26669000
H	-3.29795700	2.73742600	-1.85081200
H	-3.80932900	2.21584200	-3.46067200
C	-0.50472900	2.82197200	-2.53392900
H	-0.47154000	3.80315800	-3.01988600
H	0.47247000	2.34790400	-2.68420500
H	-0.63601000	2.98981500	-1.45859800
C	-2.89882900	-0.73953300	-3.17877200
H	-2.52947600	-1.76084500	-3.00913800
C	-4.24783300	-0.60348800	-2.47098100
H	-4.95241800	-1.34232700	-2.86837700
H	-4.69274600	0.38443300	-2.62148100
H	-4.15875500	-0.77201600	-1.39044400
C	-2.99562400	-0.50837600	-4.68562000
H	-3.68649400	-1.23671200	-5.12400700
H	-2.03058100	-0.62928800	-5.19018800
H	-3.38187100	0.48790800	-4.92250300
C	3.09148200	-0.41784500	1.37676300
C	4.19281100	0.09177700	2.05629900
H	4.84387700	0.80239100	1.55564900
C	4.46426400	-0.33454400	3.34962100
H	5.33934500	0.03965800	3.87039900
C	3.62257200	-1.26730600	3.96034100
H	3.84567600	-1.61953700	4.96302000
C	2.49809000	-1.75005800	3.30712000
H	1.85234700	-2.46825800	3.80201600

C	2.18647000	-1.32299600	2.00609500
C	0.00056600	-2.52839900	1.85398500
H	-0.18718200	-2.33341500	2.92010300
H	0.27182800	-3.58924800	1.75356100
C	-1.66131000	-3.67853700	-0.18075000
H	-1.49492300	-4.53322700	0.48914900
C	-0.50297700	-3.60557300	-1.17409400
H	-0.47112500	-4.51531600	-1.78346000
H	0.47398700	-3.50195500	-0.68730200
H	-0.63210500	-2.75585300	-1.85463900
C	-3.00153300	-3.86547100	-0.88566000
H	-2.93085000	-4.69716800	-1.59542600
H	-3.29497500	-2.97345600	-1.44700000
H	-3.80869000	-4.10355200	-0.18829500
C	-2.89880000	-2.38296600	2.23119300
H	-2.52968500	-1.72470600	3.03042000
C	-2.99535200	-3.80317500	2.78533000
H	-3.68603200	-3.81822200	3.63544400
H	-2.03020900	-4.17946000	3.14231400
H	-3.38176200	-4.50690300	2.04145600
C	-4.24793800	-1.83862100	1.75922200
H	-4.95238000	-1.81298100	2.59788100
H	-4.69286500	-2.46345700	0.97935500
H	-4.15916900	-0.81881200	1.36428200
U	0.53792800	0.00029100	-0.00063700
Co	-1.46017700	0.00035500	0.00080000

5. References

1. G. M. Sheldrick, Crystal structure refinement with SHELXL. *Acta Crystallogr. Sect. C* **71**, 3–8 (2015).
2. O. V. Dolomanov, L. J. Bourhis, R. J. Gildea, J. A. K. Howard, H. Puschmann, OLEX2: A complete structure solution, refinement and analysis program. *J. Appl. Crystallogr.* **42**, 339–341 (2009).
3. M. J. Frisch, G. W. T., H. B. Schlegel, G. E. Scuseria, M. A. Robb, J. R. Cheeseman, G. Scalmani, V. Barone, G. A. Petersson, H. Nakatsuji, X. Li, M. Caricato, A. V. Marenich, J. Bloino, B. G. Janesko, R. Gomperts, B. Mennucci, H. P. Hratchian, J. V. Ortiz, A. F. Izmaylov, J. L. Sonnenberg, D. Williams-Young, F. Ding, F. Lipparini, F. Egidi, J. Goings, B. Peng, A. Petrone, T. Henderson, D. Ranasinghe, V. G. Zakrzewski, J. Gao, N. Rega, G. Zheng, W. Liang, M. Hada, M. Ehara, K. Toyota, R. Fukuda, J. Hasegawa, M. Ishida, T. Nakajima, Y. Honda, O. Kitao, H. Nakai, T. Vreven, K. Throssell, J. A. Montgomery, Jr., J. E. Peralta, F. Ogliaro, M. J. Bearpark, J. J. Heyd, E. N. Brothers, K. N. Kudin, V. N. Staroverov, T. A. Keith, R. Kobayashi, J. Normand, K. Raghavachari, A. P. Rendell, J. C. Burant, S. S. Iyengar, J. Tomasi, M. Cossi, J. M. Millam, M. Klene, C. Adamo, R. Cammi, J. W. Ochterski, R. L. Martin, K. Morokuma, O. Farkas, J. B. Foresman, and D. J. Fox, Gaussian 16, Revision C.01. (Wallingford, CT, 2016).
4. F. Aquilante, J. Autschbach, A. Baiardi, S. Battaglia, V. A. Borin, L. F. Chibotaru, I. Conti, L. De Vico, M. Delcey, I. Fdez. Galván, N. Ferré, L. Freitag, M. Garavelli, X. Gong, S. Knecht, E. D. Larsson, R. Lindh, M. Lundberg, P. Å. Malmqvist, A. Nenov, J. Norell, M. Odelius, M. Olivucci, T. B. Pedersen, L. Pedraza-González, Q. M. Phung, K. Pierloot, M. Reiher, I. Schapiro, J. Segarra-Martí, F. Segatta, L. Seijo, S. Sen, D.-C. Sergentu, C. J. Stein, L. Ungur, M. Vacher, A. Valentini, V. Veryazov, Modern quantum chemistry with [Open]Molcas. *J. Chem. Phys.* **152**, 214117 (2020).
5. C. Adamo, V. Barone, Toward reliable density functional methods without adjustable parameters: The PBE0 model. *J. Chem. Phys.* **110**, 6158-6170 (1999).
6. S. Grimme, J. Antony, S. Ehrlich, H. Krieg, A consistent and accurate ab initio

parametrization of density functional dispersion correction (DFT-D) for the 94 elements H-Pu. *J. Chem. Phys.* **132**, 154104 (2010).

7. M. Dolg, U. Wedig, H. Stoll, H. Preuss, Energy-adjusted ab initio pseudopotentials for the first row transition elements. *J. Chem. Phys.* **86**, 866-872 (1987).
8. W. Küchle, M. Dolg, H. Stoll, H. Preuss, Energy-adjusted pseudopotentials for the actinides. Parameter sets and test calculations for thorium and thorium monoxide. *J. Chem. Phys.* **100**, 7535-7542 (1994).
9. J. M. L. Martin, A. Sundermann, Correlation consistent valence basis sets for use with the Stuttgart–Dresden–Bonn relativistic effective core potentials: The atoms Ga–Kr and In–Xe. *J. Chem. Phys.* **114**, 3408-3420 (2001).
10. X. Cao, M. Dolg, H. Stoll, Valence basis sets for relativistic energy-consistent small-core actinide pseudopotentials. *J. Chem. Phys.* **118**, 487-496 (2003).
11. X. Cao, M. Dolg, Segmented contraction scheme for small-core actinide pseudopotential basis sets. *J. Mol. Struct. THEOCHEM* **673**, 203-209 (2004).
12. R. Ditchfield, W. J. Hehre, J. A. Pople, Self-consistent molecular-orbital methods. IX. An extended Gaussian-type basis for molecular-orbital studies of organic molecules. *J. Chem. Phys.* **54**, 724-728 (1971).
13. W. J. Hehre, R. Ditchfield, J. A. Pople, Self—consistent molecular orbital methods. XII. Further extensions of Gaussian—type basis sets for use in molecular orbital studies of organic molecules. *J. Chem. Phys.* **56**, 2257-2261 (1972).
14. P. C. Hariharan, J. A. Pople, The influence of polarization functions on molecular orbital hydrogenation energies. *Theor. Chim. Acta* **28**, 213-222 (1973).
15. M. M. Francl, W. J. Pietro, W. J. Hehre, J. S. Binkley, M. S. Gordon, D. J. DeFrees, J. A. Pople, Self-consistent molecular orbital methods. XXIII. A polarization-type basis set for second-row elements. *J. Chem. Phys.* **77**, 3654-3665 (1982).
16. M. S. Gordon, J. S. Binkley, J. A. Pople, W. J. Pietro, W. J. Hehre, Self-consistent molecular-orbital methods. 22. Small split-valence basis sets for second-row elements. *J. Am. Chem. Soc.* **104**, 2797-2803 (1982).
17. J.-X. Zhang, F. K. Sheong, Z. Lin, Superatomic ligand-field splitting in ligated gold nanoclusters. *Inorg. Chem.* **59**, 8864-8870 (2020).

18. J. Chen, L. Huang, L. Wu, Y. Zhang, R. Zhang, Y. Li, Y. Zhao, L. Wang, D. Feng, M. Kira, Z. Lin, Z. Li, Isolable tetragold(0) clusters with polarity-tunable exo-Au−Au bond via intramolecular σ-aromatization. *Angew. Chem. Int. Ed.* **62**, e202311230 (2023).

19. F. K. Sheong, J.-X. Zhang, Z. Lin, Fragment aligned molecular orbital analysis: An innovative tool for analyzing atypical chemical bonding. *J. Chem. Theory Comput.* **20**, 6569-6577 (2024).

20. J.-X. Zhang, F. K. Sheong, Z. Lin, Unravelling chemical interactions with principal interacting orbital analysis. *Chem. Eur. J.* **24**, 9639-9650 (2018).

21. J.-X. Zhang, F. K. Sheong, Z. Lin, Principal interacting orbital: A chemically intuitive method for deciphering bonding interaction. *WIREs Comput. Mol. Sci.* **10**, e1469 (2020).

22. R. Krishnan, J. S. Binkley, R. Seeger, J. A. Pople, Self-consistent molecular orbital methods. XX. A basis set for correlated wave functions. *J. Chem. Phys.* **72**, 650-654 (1980).

23. A. D. McLean, G. S. Chandler, Contracted Gaussian basis sets for molecular calculations. I. Second row atoms, Z=11–18. *J. Chem. Phys.* **72**, 5639-5648 (1980).

24. A. V. Marenich, C. J. Cramer, D. G. Truhlar, Universal solvation model based on solute electron density and on a continuum model of the solvent defined by the bulk dielectric constant and atomic surface tensions. *J. Phys. Chem. B* **13**, 6378-6396 (2009).

25. B. O. Roos, R. Lindh, P.-Å. Malmqvist, V. Veryazov, P.-O. Widmark, Main group atoms and dimers studied with a new relativistic ANO basis set. *J. Phys. Chem. A* **108**, 2851-2858 (2004).

26. B. O. Roos, R. Lindh, P.-Å. Malmqvist, V. Veryazov, P.-O. Widmark, New relativistic ANO basis sets for transition metal atoms. *J. Phys. Chem. A* **109**, 6575-6579 (2005).

27. B. O. Roos, R. Lindh, P.-Å. Malmqvist, V. Veryazov, P.-O. Widmark, New relativistic ANO basis sets for actinide atoms. *Chem. Phys. Lett.* **409**, 295-299 (2005).

28. P.-O. Widmark, P.-Å. Malmqvist, B. O. Roos, Density matrix averaged atomic natural orbital (ANO) basis sets for correlated molecular wave functions. I. First row atoms. *Theor. Chim. Acta* **77**, 291-306 (1990).

29. Q. Wang, J. Zou, E. Xu, P. Pulay, S. Li, Automatic construction of the initial orbitals for efficient generalized valence bond calculations of large systems. *J. Chem. Theory Comput.* **15**, 141-153 (2019).

30. J. Zou, K. Niu, H. Ma, S. Li, W. Fang, Automatic selection of active orbitals from generalized valence bond orbitals. *J. Phys. Chem. A* **124**, 8321-8329 (2020).

31. T. A. K. Roy Dennington, and John M. Millam, GaussView, Version 6.1. *Semichem Inc., Shawnee Mission, KS, 2016.*

32. I. Mayer, P. Salvador, Overlap populations, bond orders and valences for 'fuzzy' atoms. *Chem. Phys. Lett.* **383**, 368-375 (2004).

33. I. Mayer, Charge, bond order and valence in the AB initio SCF theory. *Chem. Phys. Lett.* **97**, 270-274 (1983).
